# The genomic landscape of Nepalese Tibeto-Burmans reveals new insights into the recent peopling of Southern Himalayas

**DOI:** 10.1038/s41598-017-15862-z

**Published:** 2017-11-14

**Authors:** Guido A. Gnecchi-Ruscone, Choongwon Jeong, Sara De Fanti, Stefania Sarno, Michela Trancucci, Davide Gentilini, Anna M. Di Blasio, Mingma G. Sherpa, Phurba T. Sherpa, Giorgio Marinelli, Marco Di Marcello, Luca Natali, Davide Peluzzi, Davide Pettener, Anna Di Rienzo, Donata Luiselli, Marco Sazzini

**Affiliations:** 1 Laboratory of Molecular Anthropology & Centre for Genome Biology, Dept. of Biological, Geological and Environmental Sciences, University of Bologna, Bologna, Italy; 2 Department of Human Genetics, University of Chicago, Chicago, United States; 3 Center for Biomedical Research & Technologies, Italian Auxologic Institute IRCCS, Milan, Italy; 4 Mount Everest Summitter’s Club, Rolwaling, Dolakha, Nepal; 5 Explora Nunaat International, Montorio al Vomano, Teramo, Italy; 6 Italian Institute of Human Paleontology, Rome, Italy; 7 Present Address: Department of Archaeogenetics & Eurasia3angle research group, Max Planck Institute for the Science of Human History, Jena, Germany

## Abstract

While much research attention has focused on demographic processes that enabled human diffusion on the Tibetan plateau, little is known about more recent colonization of Southern Himalayas. In particular, the history of migrations, admixture and/or isolation of populations speaking Tibeto-Burman languages, which is supposed to be quite complex and to have reshaped patterns of genetic variation on both sides of the Himalayan arc, remains only partially elucidated. We thus described the genomic landscape of previously unsurveyed Tibeto-Burman (i.e. Sherpa and Tamang) and Indo-Aryan communities from remote Nepalese valleys. Exploration of their genomic relationships with South/East Asian populations provided evidence for Tibetan admixture with low-altitude East Asians and for Sherpa isolation. We also showed that the other Southern Himalayan Tibeto-Burmans derived East Asian ancestry not from the Tibetan/Sherpa lineage, but from low-altitude ancestors who migrated from China plausibly across Northern India/Myanmar, having experienced extensive admixture that reshuffled the ancestral Tibeto-Burman gene pool. These findings improved the understanding of the impact of gene flow/drift on the evolution of high-altitude Himalayan peoples and shed light on migration events that drove colonization of the southern Himalayan slopes, as well as on the role played by different Tibeto-Burman groups in such a complex demographic scenario.

## Introduction

To date, a great effort was devoted to investigating the origins of Asian populations dwelling immediately North of the Himalayas, especially Tibetans, to infer the demographic, biological and cultural processes that enabled human colonization of the highest plateau in the world. Archeological^1–3^ and genetic^4–6^ evidence do not rule out the possibility of Paleolithic occupation of some Tibetan regions, while the establishment of stable high-altitude settlements seems to have occurred only after the Last Glacial Maximum^2^. This suggests that the arrival of farmers descending from the proto-Tibeto-Burman Di-Qiang tribe played as the breakthrough in the successful human diffusion across the plateau^7–9^.

Accordingly, Neolithic demic movements that involved ancestors of populations speaking Tibeto-Burman languages plausibly represent the main prehistoric events having laid the foundation for the anthropological picture observable on the Tibetan plateau and in the surrounding Himalayan regions. Moreover, the more recent history of migrations, admixture and/or geographical and cultural isolation of Tibeto-Burmans, which has been not fully elucidated so far, is supposed to be quite complex and to have further reshuffled patterns of genetic variation in such a geographical area^10,11^. Mitochondrial DNA (mtDNA), Y-chromosome and linguistic data support a common origin of Tibeto-Burmans^12–15^, but provide only a limited resolution for the reconstruction of the historical processes that led these populations to be so geographically scattered. Their current distribution encompasses Tibet and other regions in China, Nepal, Bhutan, Bangladesh, Myanmar, Northeastern India and Indochina, with high cultural heterogeneity^16,17^.

Although the Himalayas were considered to be an almost insurmountable barrier to gene flow^18–20^, the presence of Tibeto-Burmans in Northeastern India and Nepal could indicate migrations that plausibly originated on the Tibetan plateau and crossed the cordillera. Unfortunately, only a couple of studies have generated genome-wide data for Tibeto-Burman populations residing South of the Himalayan arc, and they were not aimed at disentangling the overall genetic history of such a heterogeneous population group^21,22^. For instance, few genomic datasets are available for the Tibeto-Burman Sherpas^23–25^ and none for the Tamang groups that live on the southern slopes of the Himalayas or in the high-altitude transverse valleys that connect them to Tibet. Interestingly, their integration within the melting pot of Nepalese populations could be considered as the most iconic example of the mosaic of human groups with divergent biological/cultural legacies, but coexisting in a restricted geographical area, that characterizes the regions immediately South of the Himalayas. Historical records suggest that Sherpas first moved from Eastern to Central Tibet and then, approximately five hundred years ago, to the previously uninhabited high-altitude Nepalese valleys of Khumbu^26^. Their close affinity to Tibetans is well-established from both uniparental and genome-wide perspectives^9,23–27^. Nevertheless, studies based on these different sets of genetic markers drew contrasting conclusions with regard to which of these groups represents the more recently derived lineage^23,27^. Conversely, little is known about the history of Tamangs^28^, except that they show greater affinity to Tibetans based on Y-chromosome lineages^14,19,29^ compared to mtDNA ones^20^. They are thus proposed to have derived from male-biased migrations originated in a putative Tibetan homeland followed by admixture with non-Tibetan females. Whether they share recent ancestors with the Sherpas is another question that remains to be tested from a genomic perspective; answering this question may provide a key for resolving the complex relationship between modern Tibeto-Burman populations.

**Figure 1:**
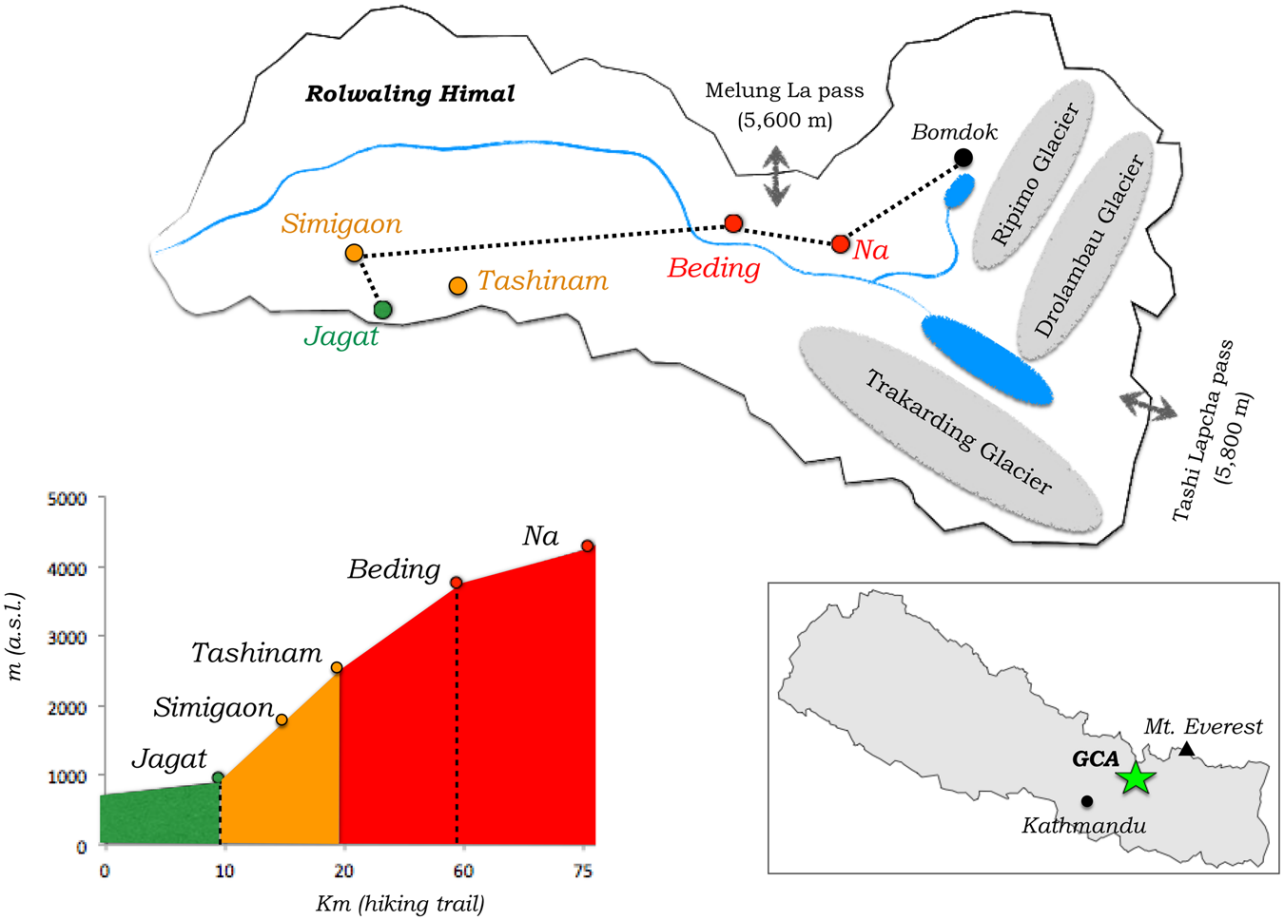
Map of the GCA and of sampling locations within and surrounding the Rolwaling Himal. On the top, the map is oriented West to East from left to right. On the bottom, the graph shows on the y-axis the elevation of the sampling locations expressed as meters above sea level (a.s.l.) and on the x-axis their distances in km of hiking trails. In the box, the location of the GCA in Nepal is specified. Bomdok is a high-altitude summer settlement at 4,900 m a.s.l. that was abandoned by the Rolwaling Sherpa community approximately 50 years ago. Both pictures are color-coded according to the ethnic groups residing in the sampling locations: green, Indo Aryan speaking groups; dark orange, Tamangs; red, Sherpas. The maps were plotted using the R software v.3.3.2 (R: A Language and Environment for Statistical Computing, R Core Team, R Foundation for Statistical Computing, Vienna, Austria (2016) https://www.R-project.org).

Therefore, the anthropological patchwork centered on Nepalese Sherpa and Tamang groups suggests that Tibeto-Burmans appreciably contributed to the complex network of migrations that characterized the peopling of the southern Himalayan slopes, which remains a largely understudied region^11,24^. Such a population picture is especially multifaceted in the Nepalese Gaurishankar Conservation Area (GCA) (Fig. 1), a number of remote Himalayan valleys that extend with the Rolwaling Himal up to the border with the Tibetan plateau. To describe the GCA genomic landscape, we generated genome-wide and/or uniparental data for 92 individuals from previously unsurveyed communities. We included people speaking Indo-Aryan languages that show cultural affinity to Indian populations and Tamangs, which spread across several GCA valleys respectively at low and medium altitudes, as well as Sherpas from the high-altitude Rolwaling valley^30^. By investigating their genomic relationships with a large set of South/East Asian (SA/EA) populations, we aimed at providing new insights on the migration and admixture events that drove the colonization of the southern slopes of the Himalayas and at dissecting the role played by different Tibeto-Burman groups in such a complex demographic scenario.

## Results

After applying stringent quality control filtering (see Materials and Methods), we generated a “GCA” dataset of 59 samples typed for 683,180 SNPs to investigate patterns of population structure and genetic diversity within the GCA. By merging it with publicly available genome-wide data (Supplementary Table S1), we assembled an “extended” dataset of 263,855 SNPs used to infer genomic relationships of GCA groups with 72 SA/EA populations.

### Setting GCA Populations into the South/East Asian Genomic Landscape

Principal Components Analysis (PCA) applied on the “GCA” dataset revealed the existence of genetically distinct populations among those sampled in the GCA. However, they do not perfectly match with broad ethnic groups recognized according to individual self-reported affiliations. In fact, while Sherpa people from the Rolwaling Himal (SRH) gathered into a single well-defined cluster, Tamangs from Tashinam (TAT) and from Simigaon (TAS) occupied different positions in the PCA space. In particular, TAS cluster was displaced toward a few Indo-Aryan speaking individuals (IAR), which were considerably scattered along PC1 (Supplementary Fig. S1). This pattern was confirmed when PCA was extended to 75 Asian populations selected from the “extended” dataset (Supplementary Fig. S2 and Supplementary Information). The sole exception to the North-South gradient observed along continental East Asia according to PC2 was represented by the outlier position occupied by high-altitude Himalayan populations, such as Tibetans (TIB) and Sherpas, as already described by several studies^8,9,23,24^.

In detail, SRH spread along the Sherpa cline together with samples from Khumbu^23^ above the TIB cluster, while TAT and, especially, TAS skew from EA cline towards the SA cluster. This trend was even more pronounced for IAR, which were substantially scattered, especially along PC1 (Supplementary Information).

### Complex Admixture Patterns of GCA and Tibeto-Burman Populations

**Figure 2:**
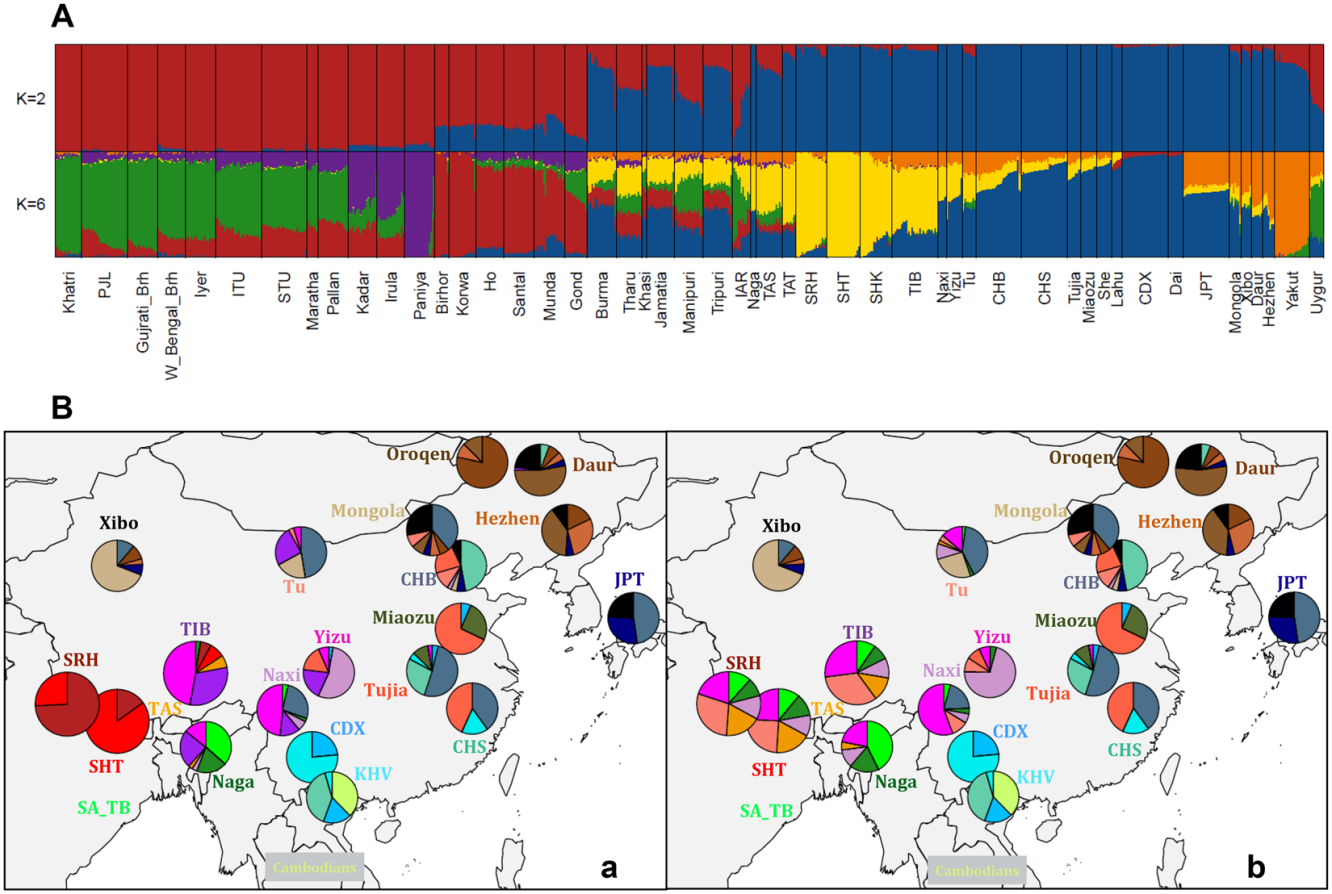
ADMIXTURE testing K = 2 and K = 6 ancestral populations and ancestry proportions of high-altitude Himalayan groups inferred by CHROMOPAINTER analyses. (**A**) ADMIXTURE at K = 2 (top) identified the EA (blue) and SA (red) ancestry components. At K = 6 (bottom), the three main SA ancestry components identified by Basu *et al*. ^12^ are appreciable, together with three EA components: the North Indian one (green), the Dravidian one (purple), the Austro-Asiatic one (red), the South EA one (blue), the North EA one (orange), as well as the “Sherpa-like” one (yellow). (**B**) Ancestry proportions of the three Himalayan populations (TIB, SRH, and SHT) inferred with modified GLOBETROTTER functions from CHROMOPAINTER outputs. (**a**) Map showing results of case *a)* obtained by searching the best matching DNA segments from individuals of each population (“recipients”) in every other individual from all populations (“donors”). (**b**) Map showing results of case *b)*, in which we excluded TIB, SRH and SHT from the donor populations in order to remove the hypothesized drift effect. In both cases, “self-copy” was allowed for all EA populations, while in case *b)* Himalayan populations could not “self-copy” or copy from each other since they were excluded from the “donors” in the upstream CHROMOPAINTER run. Population labels are color-coded according to the colours of the pie charts and, when possible, are positioned on the map according to their approximate geographic location. Pies charts representing inferred ancestry proportions for all EA populations are shown, with the exception of TAS, Cambodians and SA_TB (i.e. Jamatia, Manipuri, Tripuri, Tharu and Burma were considered together as SA Tibeto-Burmans) for simplicity since these groups are admixed with SA populations. The maps were plotted using the R software v.3.3.2 (R: A Language and Environment for Statistical Computing, R Core Team, R Foundation for Statistical Computing, Vienna, Austria (2016) https://www.R-project.org).

ADMIXTURE analysis (Fig. 2A) used to infer ancestry proportions of each GCA subject together with a large set of SA/EA samples overall agrees with PCA results. TAS were characterized by evident SA/EA admixture, TAT by a considerably reduced proportion of SA ancestry, and SRH by almost no signatures of recent SA admixture. Moreover, ADMIXTURE confirmed that IAR samples did not represent a genetically homogeneous group and were thus excluded in subsequent population-based analyses (Supplementary Information).

As previously reported^23–25^, Sherpas were found to be enriched in a specific ancestry component that in our study became appreciable for K values ≥ 4 (Fig. 2A). This component reached a proportion of 100% in almost all Sherpa individuals from Thame (SHT), a mean value of 94% in SRH and of around 80% in those from Khumjung (SHK). At K = 6, when North EA and South EA signatures differentiated and the model achieved the best predictive accuracy (Supplementary Fig. S4), this “Sherpa-like” component maintained relatively high proportions also in other Tibeto-Burmans, being instead detected at substantially lower values in several non-Tibeto-Burman EA populations (Supplementary Information).

We then computed *f3* statistics by considering all EA/SA possible population pairs of the “extended” dataset as proxies for the true ancestral admixing populations (Supplementary Table S2). This enabled us to validate the occurrence of admixture events involving both SA and EA ancestry components in almost all Tibeto-Burman populations residing South of the Himalayas (e.g. Burma, Tharu, Tripuri, Jamatia, Manipuri, TAS). The sole exceptions were represented by TAT, Nagas, SRH and SHT. Significantly negative *f3* values were obtained for SHK, suggesting admixture between either SHT or SRH and another EA or SA population.

Conversely, we observed admixture occurred in lowland Tibeto-Burman populations located North/East of the Himalayas, such as Yizu and Naxi, involving ancestral groups related to present-day TIB, Sherpas or Nagas (as one source population) and other EA populations. Moreover, high-altitude TIB also showed significant signatures of admixture involving non-admixed Sherpas (i.e. SRH and SHT) and several low-altitude EA populations.

These admixture patterns received further support from the linkage disequilibrium (LD) decay-based approach implemented in the ALDER program, which also provided time estimates for the inferred admixture events (Supplementary Table S3, Supplementary Fig. S5 and Supplementary Information).

### Disentangling the Impact of Admixture and Drift on the History of Sherpa People

A pattern of remarkable SRH intra-population homogeneity (Supplementary Information) emerged from the analyses of uniparental haplotype diversity (Supplementary Table S4 and Supplementary Table S5) and haplogroup composition (Supplementary Table S6), as well as from computation of runs of homozygosity (ROH, Supplementary Fig. S6).

This issue should be taken into account in the light of previous studies^31,32^ which showed that model-based clustering analyses are generally biased in assigning private ancestry components to highly drifted populations, as the Sherpas appears to be^23–25^. That being so, we aimed at testing whether the strong genetic drift in the Sherpa resulted in an artificial pattern of ancestry mixture in the other Tibeto-Burmans, while assigning a single “Sherpa-like” ancestry component to the Sherpa in our ADMIXTURE analysis. For this purpose, we applied a pipeline based on CHROMOPAINTER outputs as described in van Dorp *et al*.^31^ (Supplementary Information). We thus selected a subset of EA populations and conducted two separate CHROMOPAINTER runs. In the first case (a), we searched the best matching DNA segments from individuals of each population (“recipients”) in every other individual from all the considered populations (“donors”) and by allowing for “self-copy” (see Materials and Methods). Accordingly, we expected that supposed differential drift levels between Himalayan groups (i.e. TIB, SRH and SHT) would make their painting profiles substantially different. In the second case (b), we excluded these populations from the donor groups in order to remove the hypothesized drift effect and to estimate haplotype sharing of Himalayan populations with respect to all the other groups. In case a) SRH and SHT derived most of their haplotype segments from themselves (74% and 85%, respectively), while the remaining ones derived from each other (26% and 15%, respectively). On the contrary, TIB presented only 31% of “self-copy”, with the vast majority of haplotype segments being instead shared with neighbouring EA populations (i.e. 47% from Yizu, 8% from SHT, 6% from TAS, 5% from SRH and 3% from Nagas) (Fig. 2Ba). In case b) the three Himalayan groups showed extremely similar painting profiles (Fig. 2Bb), indicating that genetic drift played a substantial role in determining the results observed in case a). This pattern was further confirmed when the average length of shared haplotypes inferred by CHROMOPAINTER run (a) was calculated, showing that the Sherpa groups presented the highest (SHT) or the third highest (SRH) values among all the other EA populations included in the “extended” dataset (Supplementary Table S7).

### Genomic Relationships Between GCA and South/East Asian Populations

We used the outgroup *f3* statistics to measure shared genetic drift between GCA samples and a large set of SA/EA groups to pinpoint a plausible proxy for the ancestral population that could have introduced the EA and “Sherpa-like” genetic components in Tibeto-Burmans residing South of the Himalayas. As expected, SRH showed the highest *f3* values when compared respectively with the Sherpas from Khumbu and TIB (Supplementary Fig. S7). Moreover, the *f3* score obtained when they were tested against TIB was comparable to that found when the Tibeto-Burman Nagas from North East India were considered. Reciprocally, the same pattern was observed when TIB were contrasted to SHR and Nagas (Supplementary Fig. S7). Interestingly, the Nagas were also responsible for peak *f3* scores when both TAS and TAT were tested (Supplementary Fig. S7).

To further disentangle genomic relationships between these closely related populations, we calculated a series of *ad hoc* D-statistics by testing separately the three Tibeto-Burman GCA groups and each EA population (Supplementary Information). This confirmed that TAS and TAT were more closely related to Nagas than to TIB, while the Sherpas showed closer affinity to TIB than to any other EA population (Supplementary Fig. S8).

### The Admixed Phylogeny of Tibeto-Burman Populations

**Figure 3:**
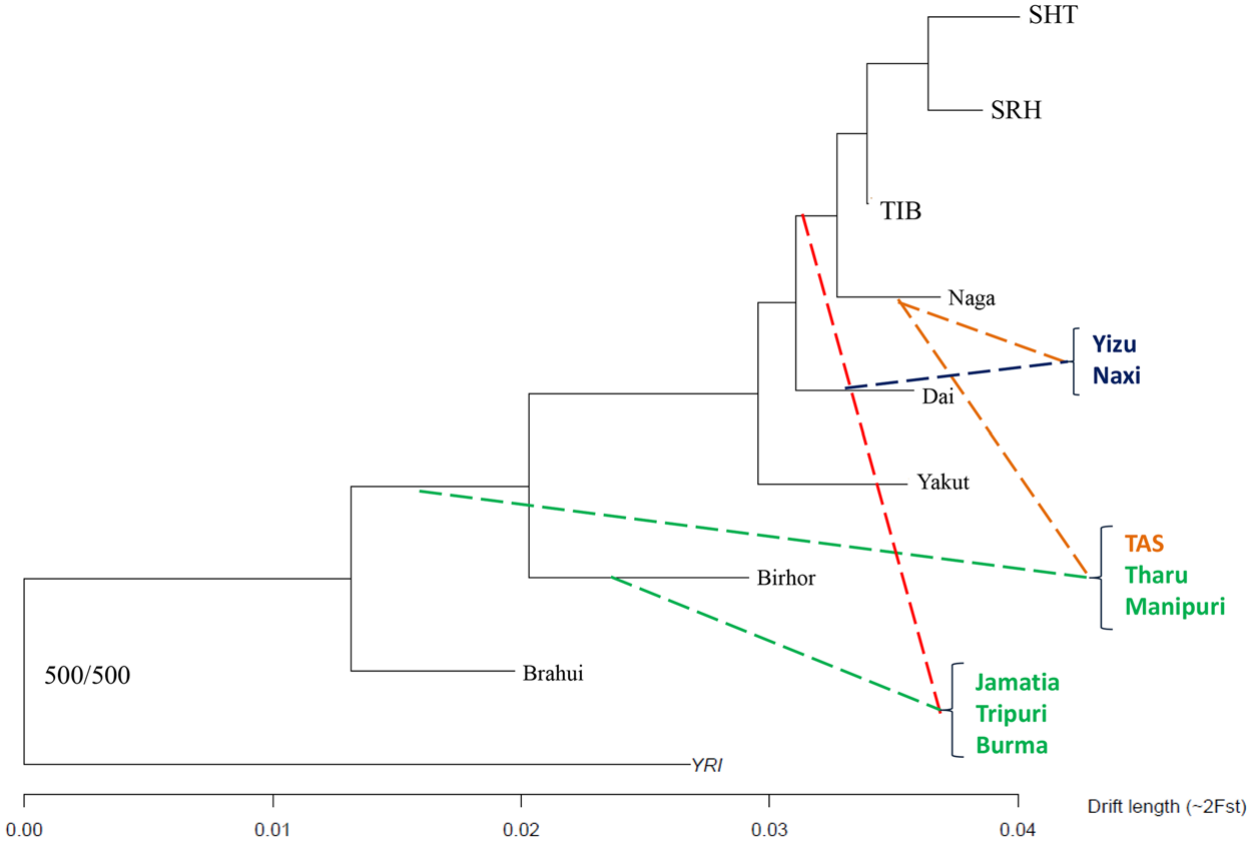
Neighbour-Joining tree reporting MIXMAPPER fitted admixture events. Scaffold phylogenetic tree of un-admixed populations with a summary of the best fits for the admixed Tibeto-Burman populations tested. Admixed populations are color-coded according to their geographic location: green (SA), orange (Nepal/North India), and blue (EA). Plots were created using the R software v.3.3.2 (R: A Language and Environment for Statistical Computing, R Core Team, R Foundation for Statistical Computing, Vienna, Austria (2016) https://www.R-project.org).

Intrigued by the presence of the “Sherpa-like” ancestry component in many EA populations and by its highest prevalence in Tibeto-Burmans from both EA and SA, we attempted to reconstruct the phylogenetic relationships of these groups by taking into account their SA/EA admixture profiles. For this purpose, we identified relatively un-admixed populations according to previously computed *f3* statistics and we used them to build a scaffold tree by means of the MIXMAPPER algorithm (Supplementary Information). The topology of the obtained tree was conserved for all the 500 bootstrap replications performed and revealed the existence of three main EA clades that mirror the EA ancestry components pointed out by ADMIXTURE analyses. The North EA-like cluster of Yakuts branched out first and then the South EA and the Tibeto-Burman clades diverged. Within this latter group, Nagas branched out before differentiation of the Sherpas from TIB (Fig. 3).

We then fitted admixed Tibeto-Burman populations on the obtained scaffold tree. Accordingly, TAS appeared to result from the admixture between a SA population and Nagas, being supported by 420 over 500 bootstrap replications. Both Yizu (500/500 replications) and Naxi (498/500 replications) were proposed to originate from the admixture between Nagas and Dai. A population located on the internal node branching after the split of Birhor from Brahui was supposed to have admixed with either Nagas or a group ancestral to the divergence between Nagas and the Tibetan/Sherpa cluster, resulting in Manipuri (491/500 replications). The same SA population could have admixed again with Nagas leading to the origin of Tharu (495/500 replications). The most frequent observation for Jamatia (314/500 replications) and Tripuri (388/500 replications) pointed them as an admixture between populations located on internal North SA nodes and a putative group occupying the internal node before the divergence between Nagas and the Tibetan/Sherpa cluster. Finally, Burma presented the same source of SA ancestry of Jamatia and Tripuri, even though their EA ancestral component had nearly the same probability to derive from the internal node of Dai (258/500 replications) or of Tibeto-Burmans (237/500 replications) (Fig. 3 and Supplementary Fig. S9).

### Isolating the East Asian Ancestry of Tibeto-Burman Populations

We applied HAPMIX to infer local ancestry and to mask the SA ancestry chunks of Tibeto-Burman groups residing South of the Himalayas. This resulted in a clear detection of their EA ancestry as tested by projecting masked haploid data in the PCA space computed on a large set of SA/EA populations (Supplementary Fig. S10).

**Figure 4:**
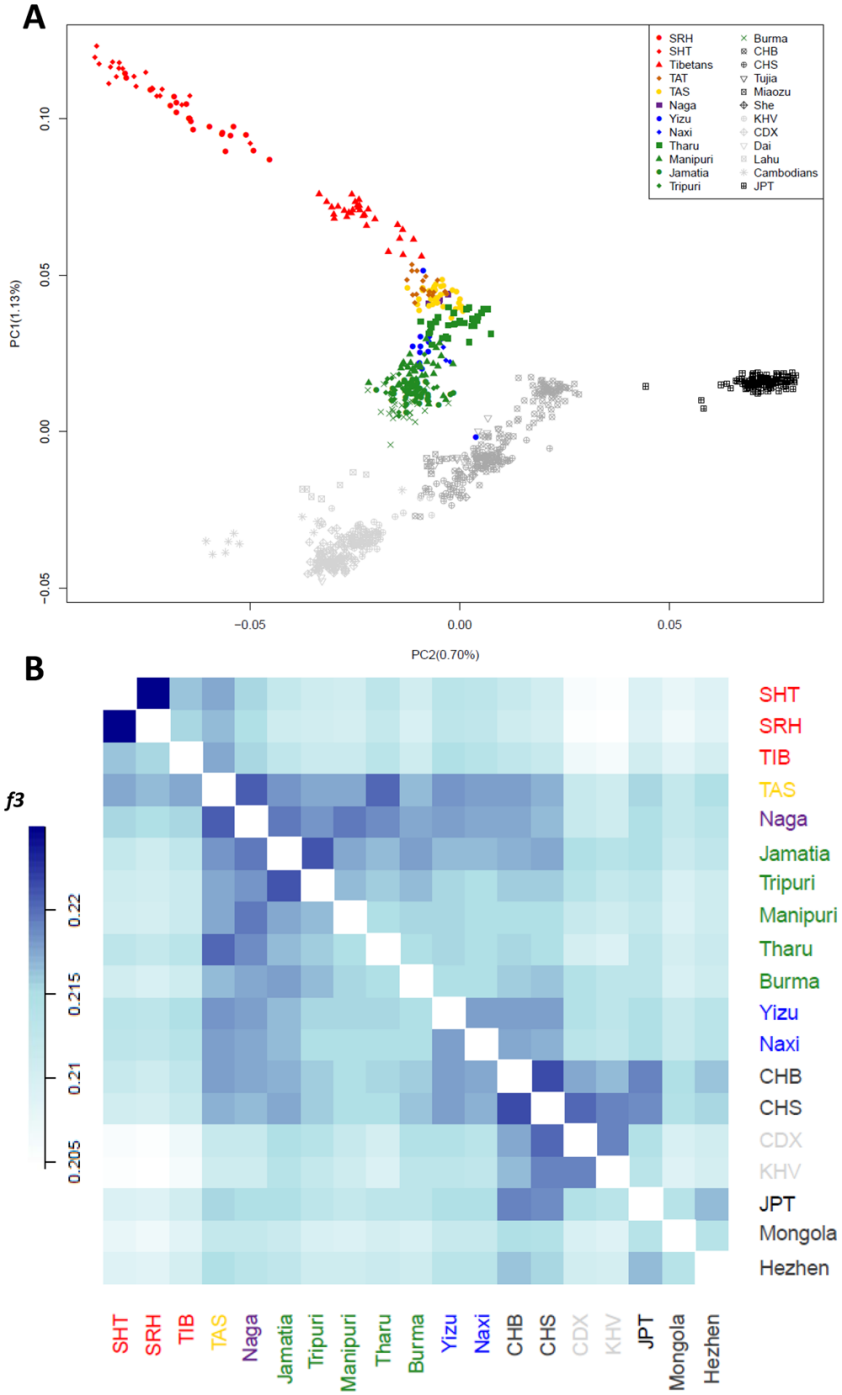
Ancestry-specific PCA and outgroup *f3* statistics computed on a subset of EA populations included in the “extended” dataset. (**A)** PCA was performed on all the 25 EA populations included in the “extended” dataset. Tibeto-Burman populations (projected into the PCA space as explained in Materials and Methods) are represented with coloured labels, whereas other EA are represented with grey-scale labels. (**B)** Outgroup *f3* statistics were performed on a subset of 18 EA populations selected from the masked “extended” dataset. Population labels are color-coded according to PCA plot. Plots were created using the R software v.3.3.2 (R: A Language and Environment for Statistical Computing, R Core Team, R Foundation for Statistical Computing, Vienna, Austria (2016) https://www.R-project.org).

To focus on the population structure within EA, we replicated PCA only on EA populations (Fig. 4A and Supplementary Information). Interestingly, Tibeto-Burmans were found to diverge from the classical North-South cline of variation present in EA, forming a distinct cluster. Moreover, Nagas, Tharu and Tamangs turned out to be located at the core of this population group from which the known Tibetan/Sherpa outlier position deviated (Fig. 4A). The remaining groups (i.e. Yizu, Naxi, Manipuri, Tripuri, Jamatia, and Burma) also diverged from such a core towards South EA populations. We can speculate that this is due to more recent gene flow between continental East Asians and several Tibeto-Burman groups, in accordance with the results obtained by MIXMAPPER analyses.

Findings from outgroup *f3* statistics computed on the masked dataset (Fig. 4B) were concordant with previous analyses and more recent genetic relationships between Tibeto-Burmans were also identified (Supplementary Information). In particular, TAS and TAT were found to be more closely related to each other (*f3* = 0.224) and then to the Nagas (*f3* = 0.221 and 0.220, respectively). This confirms their recent common ancestry and suggests that differences between them are probably due to recent geographical isolation experienced by TAT, as suggested also by their high levels of homozygosity pointed out by ROH analyses (Supplementary Fig. S6).

## Discussion

Up to date, several studies have focused on Asian populations dwelling immediately North of the Himalayas to investigate the demographic, biological, and cultural processes responsible for successful human diffusion on the Tibetan plateau^2–6^. The Himalayan arc was also proposed to have played a crucial role in shaping human population history and patterns of genetic variation in continental Asia, having acted as a geographic barrier contributing to maintain the major cultural and genetic differences observable between SA and EA populations^18–20^. Nevertheless, regions bordering the southern Himalayan slopes, together with high-altitude transverse valleys that connect them to Tibet, seem to have witnessed a few migrations of Tibeto-Burman groups that plausibly originated on the Tibetan plateau and that have then crossed the cordillera^19,24,27^. The composite anthropological picture of these southern Himalayan areas thus represents a unique example of a cultural and biological melting pot where groups speaking Indo-Aryan languages and related to the SA Hindu culture coexist with Tibeto-Burman groups of EA origins, such as the Sherpas and Tamangs.

By generating genome-wide and uniparental data for previously unsurveyed Sherpa and Tamang communities from the Nepalese GCA, we aimed at placing these populations in the context of the overall Tibeto-Burman genomic landscape to improve the reconstruction of the complex history of such a heterogeneous population group and to trace the most plausible sources of EA ancestry in SA Tibeto-Burmans. Our results thus provide the first genomic description of the above-mentioned GCA anthropological mosaic and enable us to shed new light on the colonization processes occurred on the southern slopes of the Himalayas.

### Dissecting the GCA Genomic Landscape

When contextualizing collected samples into broad patterns of Asian genetic diversity, high heterogeneity was first revealed for IAR (Supplementary Fig. S2) mainly due to extreme inter-individual variability in their SA/EA ancestry proportions (Fig. 2A). Despite admixture was proposed to be less common in IAR groups due to strict endogamous rules imposed by the caste system to Hindu populations^22,33^, this finding suggests that Indo-Aryan speaking communities residing close to the Himalayas have experienced appreciable gene flow from populations of EA origins, such as the Nepalese Tibeto-Burmans.

In contrast, the Nepalese Tibeto-Burmans appeared to have maintained remarkable internal homogeneity, with limited genetic exchange with people of SA ancestry, most pronounced in TAS. Patterns of SA/EA admixture confirmed by *f3* and ALDER approaches (Supplementary Table S2 and Supplementary Table S3) suggest that the harsh Himalayan environment has played, and still plays, an important role in limiting gene flow along a low- to high-altitude cline of geographical (more than cultural) isolation. For instance, TAS live in a village located at around 2,000 m a.s.l., but relatively well connected with low-altitude Indo Aryan settlements through a widely known trail. Accordingly, they presented more than a twofold proportion of SA admixture (23%) than TAT (9%), which instead reside at higher altitude in a more isolated valley. Such a reduced SA ancestry of TAT could explain also their lack of admixture signatures provided by the *f3* test. Despite that, ALDER succeeded in identifying admixture events in both of the Tamang communities studied here. The estimated dates of admixture ranged from 0.65 to 0.13 thousand years ago (kya), in line with the hypotheses of a recent historical arrival of the first Tamang tribes in Northern Nepal^28^. More accurate demographic tests (outgroup *f3* and D-statistics) revealed that, in addition to different admixture patterns, also a recent shared history could be ruled out for the SRH and Tamang groups, despite that they showed a similar profile of Y-chromosome lineages (Supplementary Table S6). Accordingly, we hypothesize that Tamangs reached their present-day GCA settlements along more complex migratory routes than those followed by SRH, which did not simply entail the crossing of high-altitude Tibetan/Nepalese passes. In fact, while SRH were confirmed to be genetically similar mostly to other Sherpa groups from Khumbu and then to TIB, Tamangs turned out be more closely related to the low-altitude Tibeto-Burman tribe of Nagas from Northeastern India than to Sherpas or TIB (Supplementary Fig. S7 and Supplementary Fig. S8). Interestingly, when shared genetic drift was measured between GCA samples and a large set of SA/EA groups, the Nagas were found to be responsible for top outgroup *f3* scores in almost all the performed comparisons. These findings suggest that Tamangs did not derive EA ancestry components directly from high-altitude East Asians and that this could also be true for the other Tibeto-Burman groups residing in SA. The pattern observed in the GCA is consistent with the hypothesis that the high-altitude specific genetic component remains restricted to Tibetans (e.g. those having occupied the Nepalese Mustang region, as described by Jeong *et al*.^24^) and Sherpas on the southern slopes of the Himalayas. This sheds new light on an open debate regarding the most plausible source of EA ancestry in Nepalese Tibeto-Burmans^11,19,20^. In fact, contrary to previous hypotheses^19^, our results prove that the Himalayas in most cases served as a barrier to gene flow even from North to South.

Unlike Tamangs, SRH living up to 4,180 m a.s.l. did not present any significant signature of SA admixture according to both *f3* and ALDER analyses. Moreover, they showed reduced mtDNA and Y-chromosome diversity with respect to a large set of SA/EA groups (Supplementary Table S5), together with a limited number of predominant uniparental lineages (Supplementary Table S6). All these findings are in line with those reported for the Sherpa communities of Khumbu^27^ and, coupled with remarkable autosomal homozygosity (Supplementary Fig. S6) and high values of average length of intra-population shared haplotypes (Supplementary Table S7), seem to corroborate the idea of prolonged isolation previously proposed for this ethnic group^24,25^ by historical and sociocultural studies^26,30^. Moreover, demographic inferences based on whole genome sequences of two Sherpa individuals from Khumbu^23^ showed that this group did not experience the exponential growth in effective population size having characterized the last 30 kya-history of low-altitude EA populations, but instead went through a recent bottleneck. Our CHROMOPAINTER analyses support such a scenario by showing that genetic differences between TIB, SRH and SHT are also ascribable to strong drift experienced by the Sherpas because of long-term isolation and reduced population size (Fig. 2B). In addition to this, a recent analysis of Tibetan cohorts from many sampling locations across the plateau, including the TIB sample in this study (from near Lhasa), also proved the existence of a continuum of EA/Tibetan gene flow along an East-West axis^34^_,_ which further contributed to the genetic differentiation between Tibetans and Sherpas.

### Unraveling the Genetic Legacy of Tibeto-Burman Populations

Our (Fig. 2A) and previous^23,25^ ADMIXTURE analyses identified a “Sherpa-like” ancestry component that reached 100% only in Sherpa individuals (especially SHT), but is not exclusive to Himalayan populations, being shared between several other EA and SA groups. Interestingly, most populations showing significant proportions of such a component speak Tibeto-Burman languages, with the highest values observed for Nagas and Tamangs (42%–52%), setting aside high-altitude people. We argue that these findings indicate a shared ancestry among Tibeto-Burman speaking groups, which is currently in the highest proportion in the Sherpas and more diluted in other populations due to gene flow with nearby non-Tibeto-Burman gene pools, as shown for Tibetans^34^ and for other Tibeto-Burmans in this study (Fig. 3).

However, disentangling the thread of this genetic trace is challenging as both ADMIXTURE, *f3* and ALDER tests have shown that most SA Tibeto-Burmans have experienced relatively recent and extensive gene flow from neighboring populations with SA ancestry (Supplementary Table S3). Similarly, also EA Tibeto-Burmans (e.g. Yizu and Naxi) have considerably admixed with neighboring EA populations.

To account for these gene flows, we performed a series of analyses specifically aimed at taking into account their admixed ancestry to test whether the bulk of southern Himalayan Tibeto-Burmans, in addition to Tamangs, derived EA genetic components from low-altitude populations instead that from the high-altitude Tibetan/Sherpa lineage. MIXMAPPER phylogenetic analyses first provided further evidence of a common origin of Tibeto-Burmans somewhere North of the Himalayas. Moreover, these tests showed that almost all admixed Tibeto-Burmans (from both SA and EA) are well fitted as results of admixture between Nagas (or an internal node connecting them to the Tibetan/Sherpa clade) and other populations (Fig. 3). Accordingly, we propose that the Tibetan/Sherpa and the Naga-related branches of Tibeto-Burmans split early in their history, and that, following this split, the ancestors of Nagas substantially contributed to the gene pools of many present-day populations, such as Tamangs, Naxi and Yizu. Moreover, it is noteworthy to mention that divergence between the Tibetan/Sherpa clade and Nagas cannot be explained by a single split from a common ancestor, as formally tested by computing ad hoc *f4* and D-statistics (Supplementary Information and Supplementary Table S8), suggesting that an early gene flow with non-Tibeto-Burman East Asians may have substantially contributed to it. Nevertheless, ancestry specific PCA conducted on the EA genome portions of Tibeto-Burmans identified by HAPMIX confirmed that all these groups form a cluster that departs from the known latitudinal gradient of variation characterizing continental EA (Fig. 4A). These findings were corroborated also by outgroup *f3* statistics computed on the masked dataset (Fig. 4B). In fact, they support close genetic similarity of all Tibeto-Burman populations, in line with mtDNA, Y-chromosome and linguistic evidence^12–15^, suggesting that they harbor a peculiar EA genetic makeup that was not described so far.

## Conclusions

**Figure 5:**
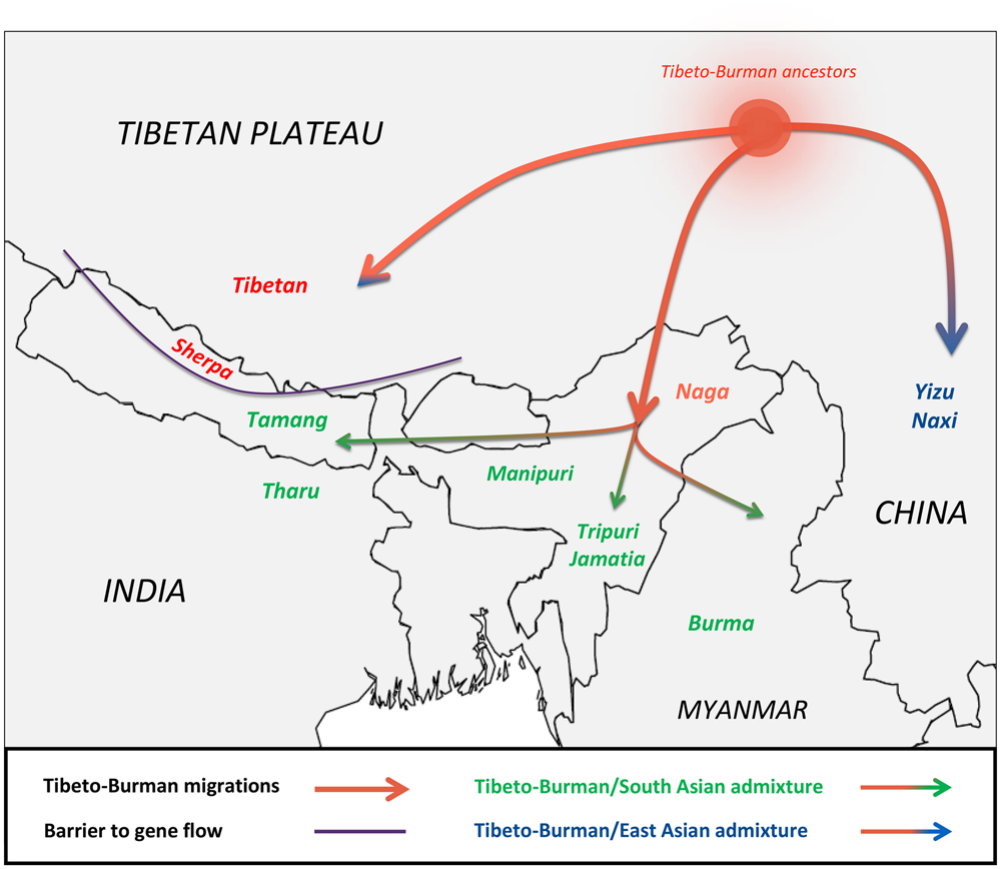
Map showing the hypothesized Tibeto-Burman migratory and admixture events as inferred by the performed analyses. Results from MIXMAPPER phylogenetic analyses and isolation of the EA genome portions of low-altitude Tibeto-Burmans via HAPMIX suggested that their peculiar EA genetic makeup derived from an ancestral gene pool plausibly originated North of the Himalayas and progressively reshaped by historical admixture with East/South Asian non-Tibeto-Burman populations along the migratory routes that diffused Tibeto-Burman ancestry components from China to Nepal across India and/or Myanmar. In fact, most Southern Himalayan Tibeto-Burmans did not derive such an EA ancestry from the high-altitude Tibetan/Sherpa lineage, in accordance with the major role played by the Himalayan arc as an almost unsurmountable barrier to gene flow. The map was plotted using the R software v.3.3.2 (R: A Language and Environment for Statistical Computing, R Core Team, R Foundation for Statistical Computing, Vienna, Austria (2016) https://www.R-project.org).

We provided new insights into the network of migration and admixture events occurred on the southern slopes of the Himalayas, thus contributing to fill, at least partially, the gap of knowledge related to such a largely understudied area. In particular, by considering a previously unsurveyed Nepalese Sherpa community that is distinct from those from the Khumbu region, we attempted to disentangle the impact of admixture and drift on the history of Himalayan populations. In fact, we found genomic evidence for their common origin, which was however followed by diverging histories characterized by variable degrees of genetic isolation and recent admixture with several sources of low-altitude EA ancestry. Nevertheless, it is noteworthy to mention that the differential levels of admixture with low-altitude East Asians observed in the examined Himalayan groups should caution about the fact that the genetic structure of high-altitude Tibetan/Sherpa populations could be more complex than previously thought. Finally, we argued that Tibeto-Burman groups residing South of the Himalayas did not derive their East Asian ancestry components from the Tibetan/Sherpa lineage, which indeed originated from a branch of ancestral Tibeto-Burmans that seems to have remained confined to high-altitude regions. Instead, the peculiar East Asian genetic makeup of Southern Himalayan Tibeto-Burman populations relates to a low-altitude gene pool for which the Nagas represent a plausible present-day proxy. This ancient genetic background was progressively reshaped by admixture events having involved a number of EA/SA populations and likely occurred along migratory routes that, by circumventing the Himalayas, diffused Tibeto-Burman ancestry components from China to Nepal across India and/or Myanmar (Fig. 5). Therefore, this study provided a step forward into the dissection of the tangled history of recent migrations, admixture and/or geographical and cultural isolation of Tibeto-Burmans, as well as into the understanding of its role in reshuffling patterns of genetic variation in the Himalayan area.

## Materials and Methods

### Samples Collection and Genotyping

Buccal swab and saliva samples analyzed in this study were collected during three scientific and humanitarian expeditions conducted in the Nepalese GCA in partnership with the ExPlora Nunaat International nonprofit organization. Such a sampling campaign focused on three main ethnic groups residing in the GCA and involved different villages of the Dolakha District distributed along a wide altitudinal range: Jagat (1,150 m a.s.l.), Simigaon (2,000 m a.s.l.), Tashinam (2,235 m a.s.l.), Beding (3,690 m a.s.l.), and Na (4,180 m a.s.l.) (Fig. 1). In particular, people speaking Indo-Aryan languages (N = 23) were sampled in Jagat, Tamangs were recruited in Simigaon (N = 26) and Tashinam (N = 11), while Sherpa samples (N = 32) were collected in Beding and Na.

Each participant compiled an ethnographic questionnaire and biological samples were collected only from subjects whose grandparents belonged to the same ethnic group. Informed consent was obtained from all participants and on 04/08/2013 the University of Bologna ethics committee released approval for the present study (within the framework of the ERC-2011-AdG 295733 project), which was designed and conducted in accordance with relevant guidelines and regulations and according to ethical principles for medical research involving human subjects stated by the WMA Declaration of Helsinki.

We extracted DNA from buccal swab via a Salting Out modified protocol and from saliva samples collected with the Oragene DNA (OG-500) kit with the *prepIT-L2P* protocol (DNA Genotek, Ottawa, Ontario, Canada). We then quantified purified DNA with the Quant-iT dsDNA Broad-Range Assay Kit (Invitrogen Life Technologies, Carlsbad, CA, USA). All the 92 collected samples were sequenced for mtDNA hypervariable regions I and II (HVSI and HVSII) by means of Sanger sequencing and using the BigDye Terminator v3.1 Cycle Sequencing Kit (Thermo Fisher Scientific, Waltham, MA, USA). A subset of 63 male individuals were also typed for 23 Y-chromosome STRs loci using the PowerPlexY23 System kit (Promega, Madison, WI, USA). Typing of uniparental markers was performed at the Molecular Anthropology Lab of the University of Bologna. 75 samples were then selected to be genotyped for ~720,000 genome-wide SNPs with the HumanOmniExpress v 1.1 chip (Illumina, San Diego, CA, USA). Genotyping was performed at facilities of the Human Genetics Dept. of the University of Chicago and at the Center for Biomedical Research & Technologies of the Italian Auxologic Institute.

### Data Curation

A detailed description of the analyses on uniparental haplotypes as well as of the quality control (QC) and merging steps on the genome-wide datasets are provided in Supplementary Information.

After QC performed with PLINK v1.07^35^, a “GCA” dataset consisting of 59 samples successfully typed for 683,180 SNPs was generated. An “extended” dataset of 263,855 SNPs was also obtained by merging GCA genotypes with publicly available genome-wide data retrieved from phase 3 of the 1000 Genomes Project^36^, the HGDP project^10^, as well as many published studies focused on SA and EA groups (Supplementary Table S2). Genotype-based population structure and admixture analyses were applied to 107,373 SNPs after LD-pruning (r^2^ < 0.2) and by removing sites with a minor allele frequency (MAF) < 0.01. Such a pruned dataset was also used to measure ROH. For these purposes, the number of genomic segments in homozygosity and their mean length were calculated with PLINK v1.07 by applying default parameters settings.

Haplotype-based population structure analyses and local ancestry inference were instead applied on the unpruned “extended” dataset. Haplotypes phasing was performed by means of the statistical-based approach implemented in SHAPEIT2 v2.r790^37^ using default parameters and HapMap phase 3 recombination maps.

### Genotype-based Population Structure Analyses

PCA was applied sequentially on the LD-pruned versions of both “GCA” and “extended” datasets to check for the presence of potential genotypes inconsistency due to errors occurred in the merging procedure, as well as on a subset of 75 Asian groups selected from the “extended” dataset. To compute PCA, we used the *smartpca* method implemented in the EIGENSOFT package v6.0.1^38^.

To obtain an overall picture of ancestry proportions for each examined genome, we applied the model-based clustering algorithm implemented in ADMIXTURE v1.22^39^ assuming K = 2 to K = 12 ancestral populations. We ran fifty replicates with different random seeds for each K to monitor for convergence and only those presenting the highest log-likelihood values were plotted. Concurrently, we calculated cross-validation (CV) errors for each K in order to identify the most plausible number of ancestry components. Once verified that GCA samples showed no signatures of recent admixture involving ancestry sources ascribable to non-Asian populations, we replicated admixture analyses with the same procedure to test K = 2 to K = 10 putative ancestral groups on a representative subset of Asian populations.

### Tests Aimed at Providing Demographic Inferences

To formally test for admixture, we performed the three-population test (*f3*)^40^ using the ADMIXTOOLS v3.0 package^41^. Z-scores were calculated via a Block Jack-knife approach to assess test significance and potential source populations (i.e. those showing significant Z-scores ≤ −2) were submitted to further validation with ALDER v1.03^42^. This method was also used to derive the number of generations since admixture by fitting one exponential curve (i.e. assuming a single pulse of admixture) to the data. Obtained results were converted in time estimates for the inferred admixture events by assuming 25 years per generation.

To test more refined demographic hypotheses (see Results), we used ADMIXTOOLS v3.0 to measure shared genetic history between two populations via the outgroup *f3* statistics, which is less influenced than traditional *F*_ST_ by strong genetic drift occurred in one of them^43^. The same package was used to estimate population genomic distances by computing the D-statistics^44^. In both cases, YRI population was used as an outgroup, being considered as a target of admixture in the *f3* test so that high positive values were interpreted as evidence for a close relationship between the two supposed source groups. According to these approaches, we tested 72 populations selected from the “extended” dataset to identify those most closely related to GCA groups.

We further explored phylogenetic relationships between GCA populations, other Tibeto-Burman groups included in the “extended” panel, and putative source populations pointed out by the above-mentioned analyses, by taking into account recent admixture involving SA and EA groups. For this purpose, we calculated a matrix of *f2* statistics between each population pair by applying the procedure implemented in MIXMAPPER v2.0^45^ and we used it to construct a neighbor-joining tree of non-admixed Asian populations selected according to *f3* results. Supposed admixed populations were then fitted in the scaffold tree by resolving *f2* statistics between them and each of the non-admixed groups. To evaluate the robustness of the topology of scaffold trees and of the branch point for test populations, we conducted 500 bootstraps replications.

### Haplotype-based Estimates of Ancestry Proportions and Magnitude of Drift

We used CHROMOPAINTERv2^46^ to “paint” haplotypes of single individuals as mosaics of all other individuals’ haplotypes observed in the dataset. For each of the performed CHROMOPAINTER runs (see Results), we first estimated the mutation/emission and the switch rate parameters with 10 steps of the E-M algorithm on a subset of chromosomes {4, 10, 15, 22}. We then averaged the obtained values across chromosomes, weighted by the number of markers, and then across individuals. We finally run CHROMOPAINTER on all chromosomes using the estimated mutation/emission and switch rate parameters.

To infer individual ancestry proportions (i.e. the average proportion of the genome that each recipient population copies from the donor groups), we combined the copying length matrixes between chromosomes and we obtained an estimate of the total genomic length that each recipient individual copies from each donor population. Then we performed multiple linear regression as described in Leslie *et al*.^47^ by applying the dedicated functions implemented in GLOBETROTTER^48^ and modified as described in van Dorp *et al*.^31^ thus allowing “self-copy” between individuals belonging to the same group in order to account for intra-population haplotype sharing.

### Local Ancestry Inference

We used the algorithm implemented in HAPMIX v1.1^49^ to infer local ancestry of Tibeto-Burmans identified as admixed by assigning an ancestry probability score for each SNP. Chunks of contiguous ancestry in the haplotypes of tested admixed individuals were identified given haploid data from two sets of reference ancestral populations. We selected as reference SA and EA 1000 Genomes Project populations included in the phased “extended” dataset. We used the mean number of generations since admixture inferred with ALDER and we recovered proportions of SA and EA admixture for individuals submitted to ADMIXTURE analysis at K = 2, finally calculating the mean proportion for each of the tested groups. We then selectively masked chunks of the genome assigned to EA ancestry with a probability lower than 90%.

To validate results of local ancestry inference, we performed PCA on a set of 75 Asian populations using the *lsqproject = YES* function implemented in EIGENSOFT v6.0.1 to project Tibeto-Burman EA ancestry-specific samples (EA-TB) on the PCA space and overcoming potential bias related to the several missing data included in the EA-TB dataset. We then replicated PCA on a subset of EA populations. Finally, to test how the EA-TB relate to the other EA populations, we computed outgroup *f3* statistics as described above.

### Data Availability

The dataset generated during the current study is available at the Molecular Anthropology Lab repository, http://www.bioanthropologybologna.eu.

## Electronic supplementary material

Supplementary Information

Supplementary Tables S1-S8

## References

[1] Aldenderfer M, Yinong Z (2004). The Prehistory of the Tibetan Plateau to the Seventh Century A.D.: Perspectives and Research from China and the West Since 1950. Journal of World Prehistory.

[2] Aldenderfer M (2011). Peopling the Tibetan plateau: insights from archaeology. High Alt. Med. Biol..

[3] Rhode D (2016). A biogeographic perspective on early human colonization of the Tibetan Plateau. Archaeological Research in Asia.

[4] Su B (2000). Y chromosome haplotypes reveal prehistorical migrations to the Himalayas. Hum. Genet..

[5] Zhao M (2009). Mitochondrial genome evidence reveals successful Late Paleolithic settlement on the Tibetan Plateau. Proc. Natl. Acad. Sci. USA.

[6] Qin Z (2010). A mitochondrial revelation of early human migrations to the Tibetan Plateau before and after the last glacial maximum. Am. J. Phys. Anthropol..

[7] Zhao Y-B (2011). Ancient DNA evidence supports the contribution of Di-Qiang people to the han Chinese gene pool. Am. J. Phys. Anthropol..

[8] Qi X (2013). Genetic evidence of paleolithic colonization and neolithic expansion of modern humans on the tibetan plateau. Mol. Biol. Evol..

[9] Lu D (2016). Ancestral Origins and Genetic History of Tibetan Highlanders. Am. J. Hum. Genet..

[10] Li JZ (2008). Worldwide human relationships inferred from genome-wide patterns of variation. Science.

[11] Wang H-W (2012). Revisiting the role of the Himalayas in peopling Nepal: insights from mitochondrial genomes. J. Hum. Genet..

[12] Yao Y-G, Kong Q-P, Bandelt H-J, Kivisild T, Zhang Y-P (2002). Phylogeographic differentiation of mitochondrial DNA in Han Chinese. Am. J. Hum. Genet..

[13] Wen B (2004). Analyses of genetic structure of Tibeto-Burman populations reveals sex-biased admixture in southern Tibeto-Burmans. Am. J. Hum. Genet..

[14] Gayden T (2009). Genetic insights into the origins of Tibeto-Burman populations in the Himalayas. J. Hum. Genet..

[15] Stoneking M, Delfin F (2010). The human genetic history of East Asia: weaving a complex tapestry. Curr. Biol..

[16] van Driem, G. Neolithic correlates of ancient Tibeto-Burman migrations in *Archaeology and Language* (eds Blench, R., Spriggs, M.) 67–102 (Routledge, 1998).

[17] van Driem, G. Tibeto-Burman phylogeny and prehistory: Languages, material culture and genes in *Examining the Farming/Language Dispersal Hypothesis* (eds Bellwood, P., Renfrew, C.) 233–249 (McDonald Institute for Archaeological Research, 2002).

[18] Cordaux R (2004). Independent origins of Indian caste and tribal paternal lineages. Curr. Biol..

[19] Gayden T (2007). The Himalayas as a directional barrier to gene flow. Am. J. Hum. Genet..

[20] Gayden T (2013). The Himalayas: barrier and conduit for gene flow. Am. J. Phys. Anthropol..

[21] Chaubey G (2011). Population genetic structure in Indian Austroasiatic speakers: the role of landscape barriers and sex-specific admixture. Mol. Biol. Evol..

[22] Basu A, Sarkar-Roy N, Majumder PP (2016). Genomic reconstruction of the history of extant populations of India reveals five distinct ancestral components and a complex structure. Proc. Natl. Acad. Sci. USA.

[23] Jeong C (2014). Admixture facilitates genetic adaptations to high altitude in Tibet. Nat Commun.

[24] Jeong C (2016). Long-term genetic stability and a high-altitude East Asian origin for the peoples of the high valleys of the Himalayan arc. Proc. Natl. Acad. Sci. USA.

[25] Zhang C (2017). Differentiated demographic histories and local adaptations between Sherpas and Tibetans. Genome Biol..

[26] Oppitz, M. Myths and facts: Reconsidering some data concerning the clan history of the Sherpas (1974) Available at: http://www.dspace.cam.ac.uk/handle/1810/227209.

[27] Bhandari S (2015). Genetic evidence of a recent Tibetan ancestry to Sherpas in the Himalayan region. Sci Rep.

[28] Campbell, B. The Heavy loads of Tamang Identity in *Nationalism and Ethnicity in* Nepal (eds Gellner, D., Pfaff-Czarnecka, J., Whelpton, J.) 205–235 (Vajra Publications, 1997).

[29] Gayden T, Bukhari A, Chennakrishnaiah S, Stojkovic O, Herrera RJ (2012). Y-chromosomal microsatellite diversity in three culturally defined regions of historical Tibet. Forensic Sci Int Genet.

[30] Baumgartner, R. Farewell to Yak and Yeti: The Rolwaling Sherpas: Facing a Globalised World (Vajra Books, 2015).

[31] van Dorp L (2015). Evidence for a Common Origin of Blacksmiths and Cultivators in the Ethiopian Ari within the Last 4500 Years: Lessons for Clustering-Based Inference. PLoS Genet..

[32] Falush, D., van Dorp, L. & Lawson, D. A tutorial on how (not) to over-interpretSTRUCTURE/ADMIXTURE bar plots. *bioRxiv*10.1101/066431 (2016).

[33] Moorjani P (2013). Genetic evidence for recent population mixture in India. Am. J. Hum. Genet..

[34] Jeong C (2017). A longitudinal cline characterizes the genetic structure of human populations in the Tibetan plateau. PLoS One..

[35] Purcell S (2007). PLINK: a tool set for whole-genome association and population-based linkage analyses. Am. J. Hum. Genet..

[36] The 1000 Genomes Project Consortium. A global reference for human genetic variation. *Nature* **526**, 68–74 (2015). 10.1038/nature15393 PMC4750478 26432245

[37] Delaneau O, Zagury J-F, Marchini J (2013). Improved whole-chromosome phasing for disease and population genetic studies. Nat. Methods.

[38] Patterson N, Price AL, Reich D (2006). Population structure and eigenanalysis. PLoS Genet..

[39] Alexander DH, Novembre J, Lange K (2009). Fast model-based estimation of ancestry in unrelated individuals. Genome Res..

[40] Reich D, Thangaraj K, Patterson N, Price AL, Singh L (2009). Reconstructing Indian population history. Nature.

[41] Patterson N (2012). Ancient admixture in human history. Genetics.

[42] Loh P-R (2013). Inferring admixture histories of human populations using linkage disequilibrium. Genetics.

[43] Raghavan M (2014). Upper Palaeolithic Siberian genome reveals dual ancestry of Native Americans. Nature.

[44] Green RE (2010). A draft sequence of the Neandertal genome. Science.

[45] Lipson M (2013). Efficient moment-based inference of admixture parameters and sources of gene flow. Mol. Biol. Evol..

[46] Lawson DJ, Hellenthal G, Myers S, Falush D (2012). Inference of population structure using dense haplotype data. PLoS Genet..

[47] Leslie S (2015). The fine-scale genetic structure of the British population. Nature.

[48] Hellenthal G (2014). A genetic atlas of human admixture history. Science.

[49] Price AL (2009). Sensitive detection of chromosomal segments of distinct ancestry in admixed populations. PLoS Genet..

