# Supplementary material for: The genomic landscape of Nepalese Tibeto-Burmans reveals new insights into the recent peopling of Southern Himalayas
Source: Sci Rep. 2017 Nov 14;7:15512. doi: 10.1038/s41598-017-15862-z (PMC5686152; doi:10.1038/s41598-017-15862-z)
Supplement: Supplementary file 1 — Supplementary Information [file 41598_2017_15862_MOESM1_ESM.pdf]

The genomic landscape of Nepalese Tibeto-Burmans reveals new insights into the recent peopling of Southern Himalayas

Guido A. Gnecchi-Ruscone[1], Choongwon Jeong[2,†], Sara De Fanti[1], Stefania Sarno[1], Michela Trancucci[1], Davide Gentilini[3], Anna M. Di Blasio[3], Mingma G. Sherpa[4], Phurba T. Sherpa[4], Giorgio Marinelli[5], Marco Di Marcello[5], Luca Natali[5,6], Davide Peluzzi[5], Davide Pettener[1], Anna Di Rienzo[2#], Donata Luiselli[1*] & Marco Sazzini[1#*]

[1]Laboratory of Molecular Anthropology & Centre for Genome Biology, Dept. of Biological, Geological and Environmental Sciences, University of Bologna, Italy
[2]Department of Human Genetics, University of Chicago, United States
[3]Center for Biomedical Research & Technologies, Italian Auxologic Institute IRCCS, Milan, Italy
[4]Mount Everest Summitter's Club, Rolwaling, Dolakha, Nepal
[5]Explora Nunaat International, Montorio al Vomano, Teramo, Italy
[6]Italian Institute of Human Paleontology, Rome, Italy

[†]current address: Department of Archaeogenetics & Eurasia3angle research group, Max Planck Institute for the Science of Human History, Jena, Germany

*corresponding authors (emails: marco.sazzini2@unibo.it; donata.luiselli@unibo.it)

[#] These authors contributed equally to this work

Supplementary Information

Includes Supplementary Figures S1-S10, Legends of Supplementary Tables, Supplementary Results, Supplementary Materials and Methods, Supplementary References.

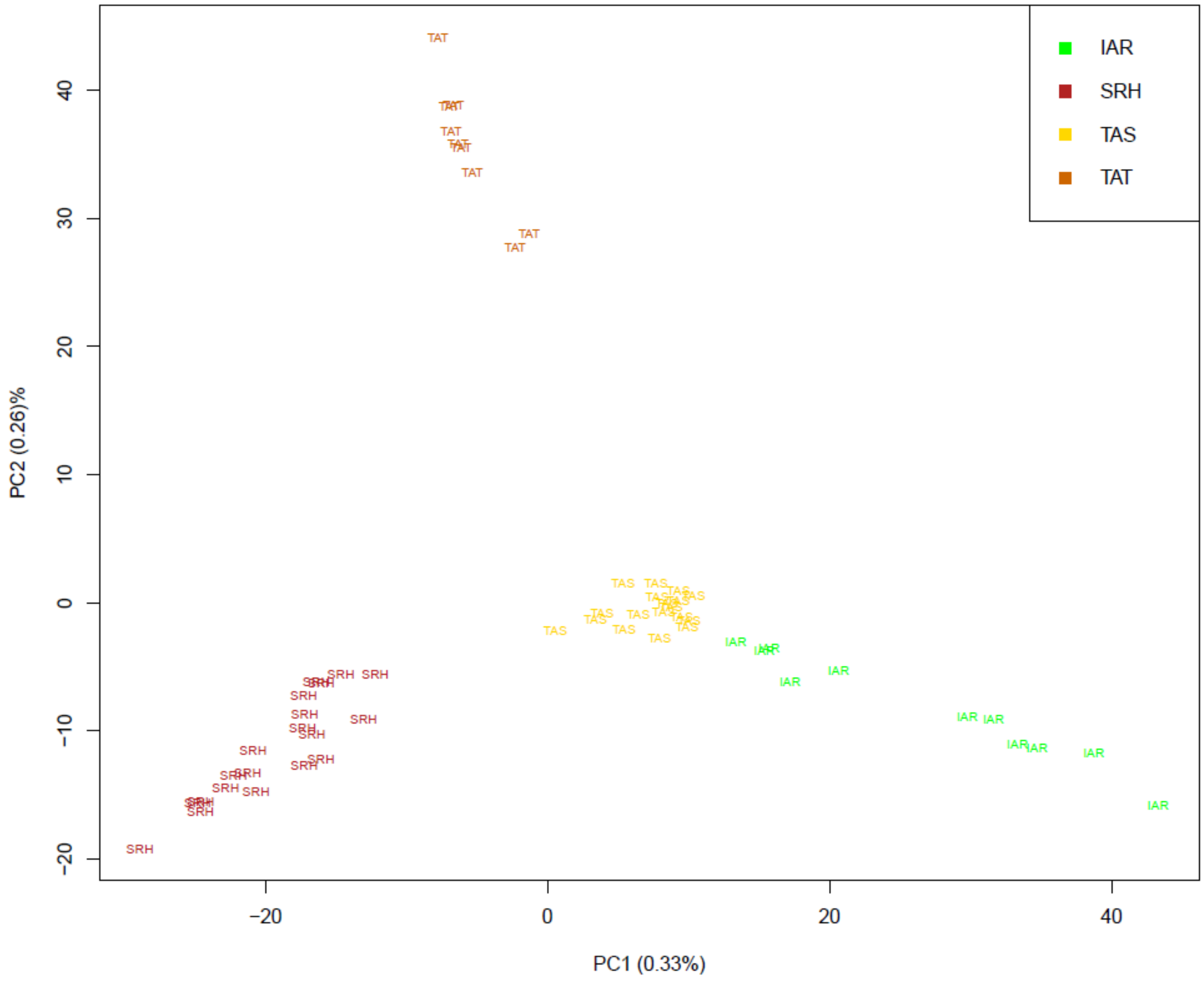

Supplementary Fig. S1. PCA performed on samples included in the "GCA" dataset. PCA was calculated after removing three individuals exceeding ± 3 standard deviations from the mean PC1 or PC2 eigenvector calculated for their respective self-assigned ethnic groups. Three main clusters reflecting the examined ethnic groups and/or sampling locations were identified: Sherpas from the Rolwaling Himal, SRH (red); Tamangs from Tashinam, TAT (brown), Tamangs from Simigaon, TAS (yellow). Conversely, Indo-Aryan speaking samples (IAR, green) could be not considered as a homogeneous population.

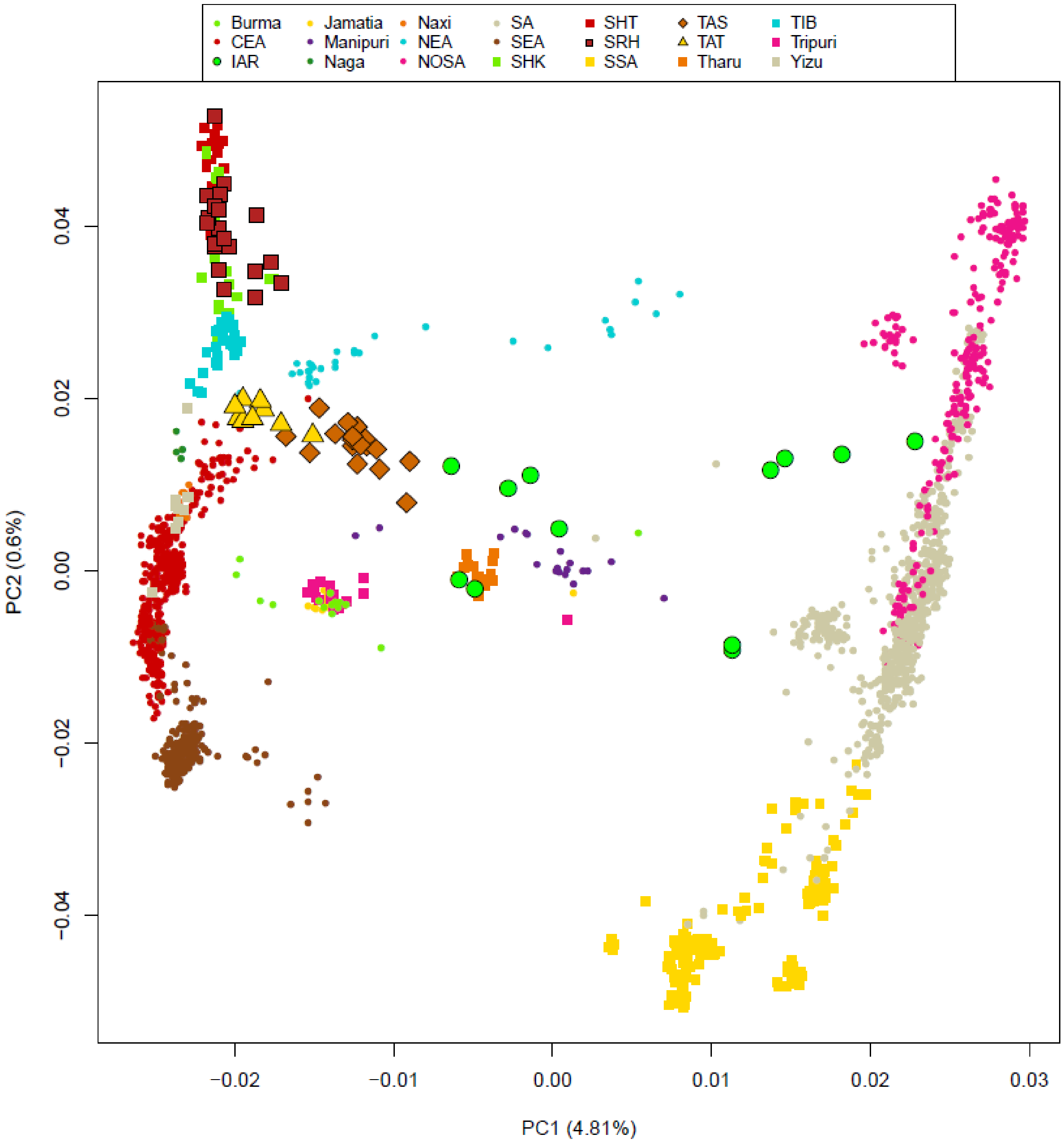

Supplementary Fig. S2. PCA performed on 75 Asian populations included in the "extended" dataset. PC1 describes genetic differentiation between EA (left) and SA (right) populations. PC2 instead shows the latitudinal gradients observable within the two macro-groups. The North to South SA gradient (top to bottom) and the North to South EA gradient (centre to bottom) are displayed together with the outlier position of Himalayan populations (Tibetans, TIB and Sherpas). Samples from the GCA populations are highlighted with bigger labels: SRH (red squares), TAT (yellow triangles), TAS (dark-orange rhombus), IAR (green circles). EA populations are labelled according to macro-groups: Central EA, CEA; North EA, NEA; North SA, NOSA; South SA, SSA.

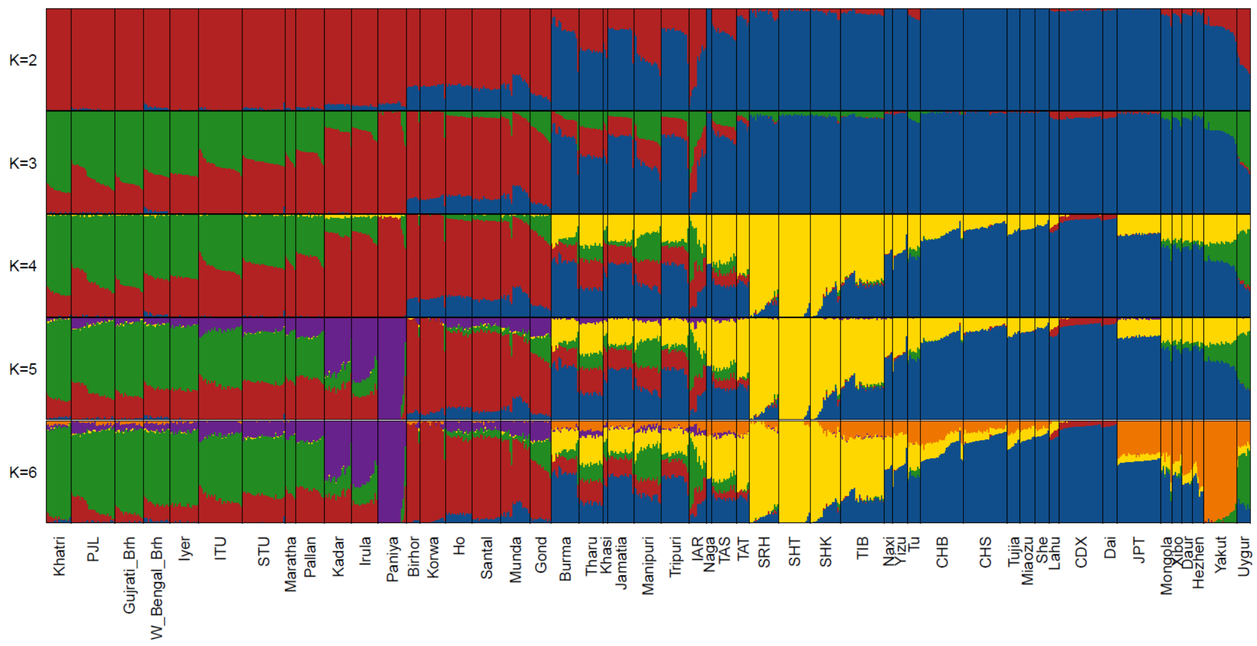

Supplementary Fig. S3. Ancestry proportions at K = 2 to K = 6 (from top to bottom) estimated with ADMIXTURE. ADMIXTURE analyses were performed on 843 individuals belonging to 50 Asian populations selected from the "extended" dataset. For each K only the runs with the highest Log-Likelihood are plotted.

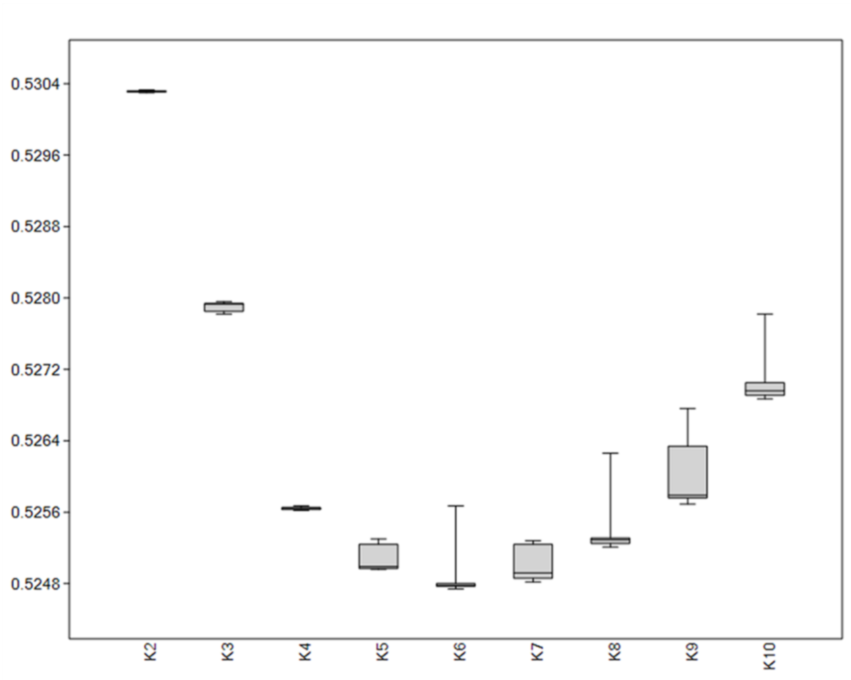

Supplementary Fig. S4. Cross-validation (CV) errors for ADMIXTURE clustering analyses. CV were computed for all the 50 runs performed for each tested K. The best predictive accuracy was achieved by the model when six ancestral components (K = 6) were tested.

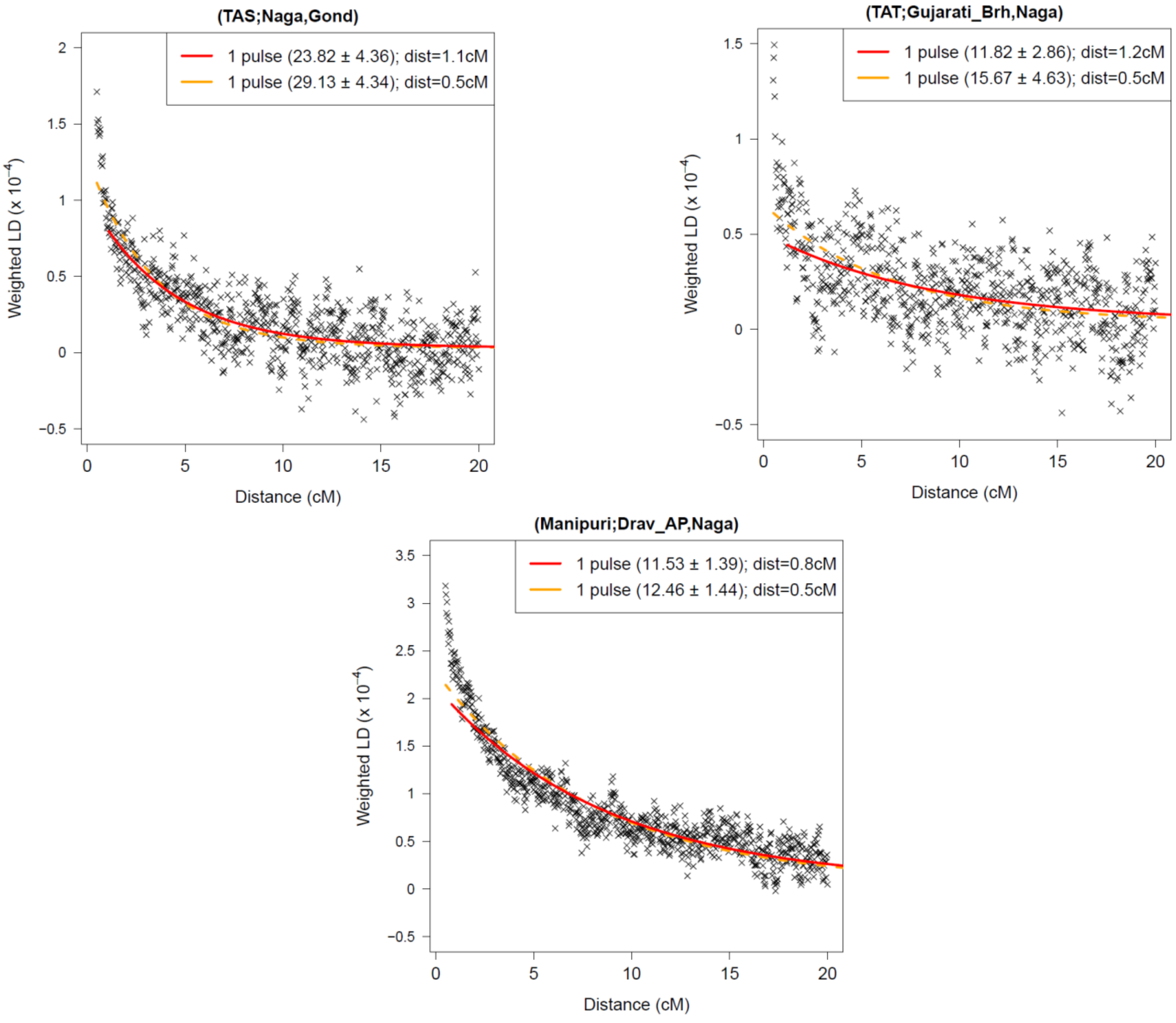

Supplementary Fig. S5. Weighted LD curves as function of genetic distance (cM) obtained with ALDER. We report three LD curves as examples of the several performed ALDER tests (Supplementary Table S3). In particular, they describe in turn TAS (top left), TAT (top right) and Manipuri (bottom) as the admixed groups, as well as Nagas as the EA proxy source group and a SA population as the other source. Even though these tests resulted highly significant (Supplementary Table S3), the curve related to TAT appears to be more noisy, probably due to the lower sample sizes of this group (N = 9) and/or to the lower SA contribution (9%) to their ancestry.

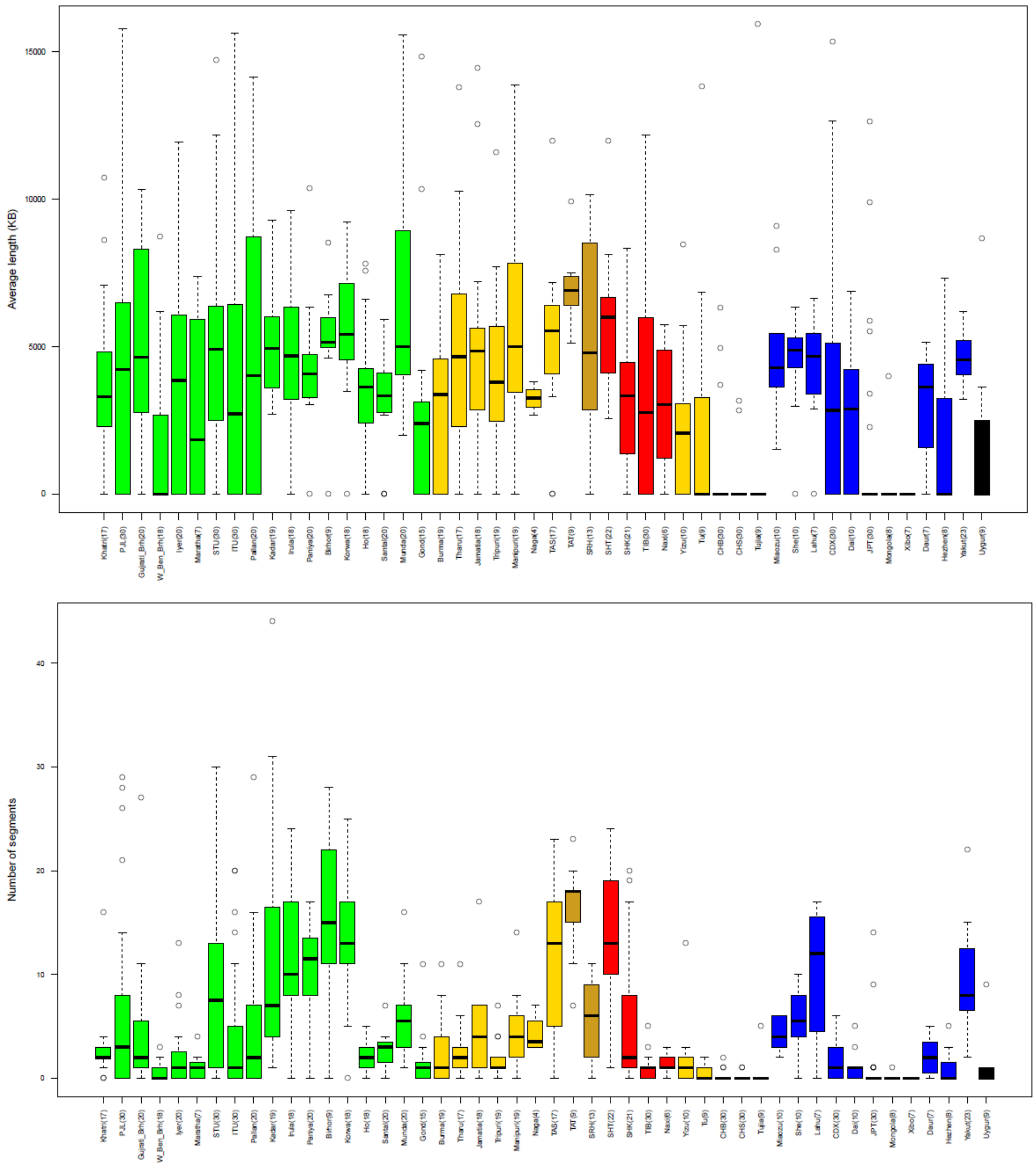

Supplementary Fig. S6. ROH performed on a subset of Asian populations included in the "extended" dataset. The top panel shows the average length of segments in homozygosity, while the bottom panel shows the total number of segments in homozygosity. In brackets, sample sizes are reported for each population. Bar plots are color-coded according to the following groups: green (SA), black (North EA), blue (EA), yellow (Tibeto-Burmans), dark-yellow (Tamangs), red (TIB and Sherpas).

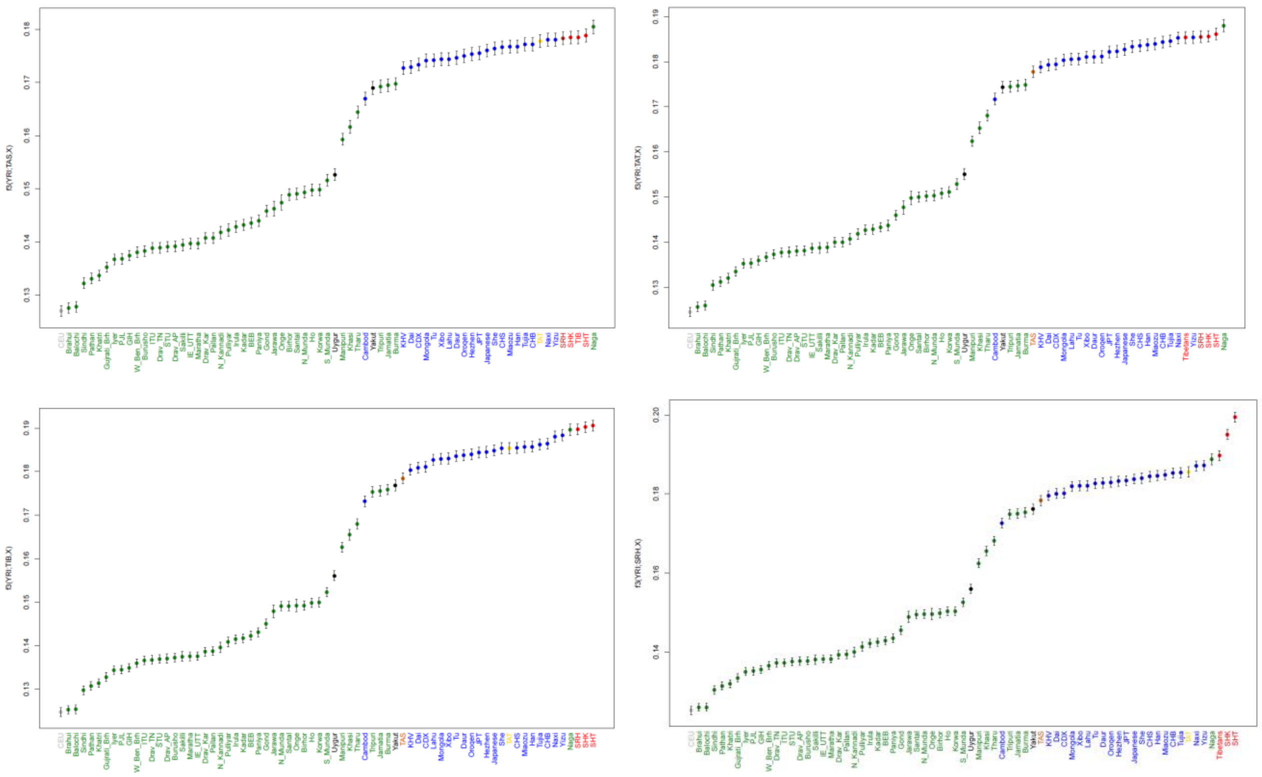

Supplementary Fig. S7. Outgroup *f3* statistics measuring the shared genetic drift between GCA samples and a large set of SA/EA groups. The analyses were performed with the *qp3Pop* function implemented in ADMIXTOOLS on the 75 Asian populations included in the “extended” dataset. YRI were used as outgroup population for every test. Populations are color-coded according to the following groups: grey (CEU), green (SA), blue (EA), black (North EA), yellow (TAS), dark-orange (TAT), red (TIB and Sherpas).

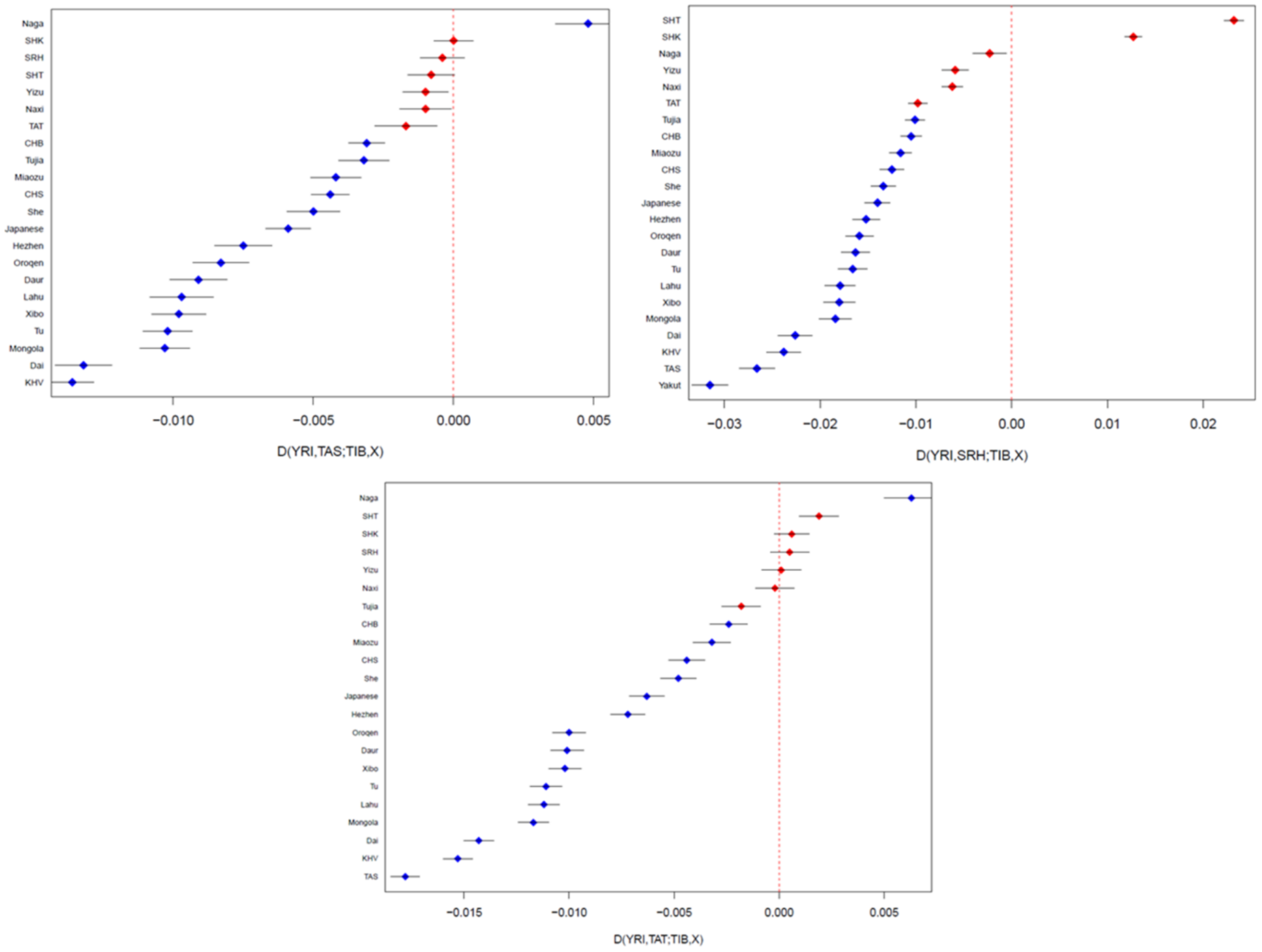

Supplementary Fig. S8. D-statistics computed for the GCA groups. The analyses were performed with the *qpDstat* function implemented in ADMIXTOOLS. On the y-axis are located the EA populations included in the "extended" dataset and used in turn for testing each GCA group. EA Tibeto-Burman populations are displayed in red, while all the other populations are coloured in blue. Negative D values indicate closer affinity of the tested GCA group with TIB, whereas positive values indicate closer affinity with the considered EA population.

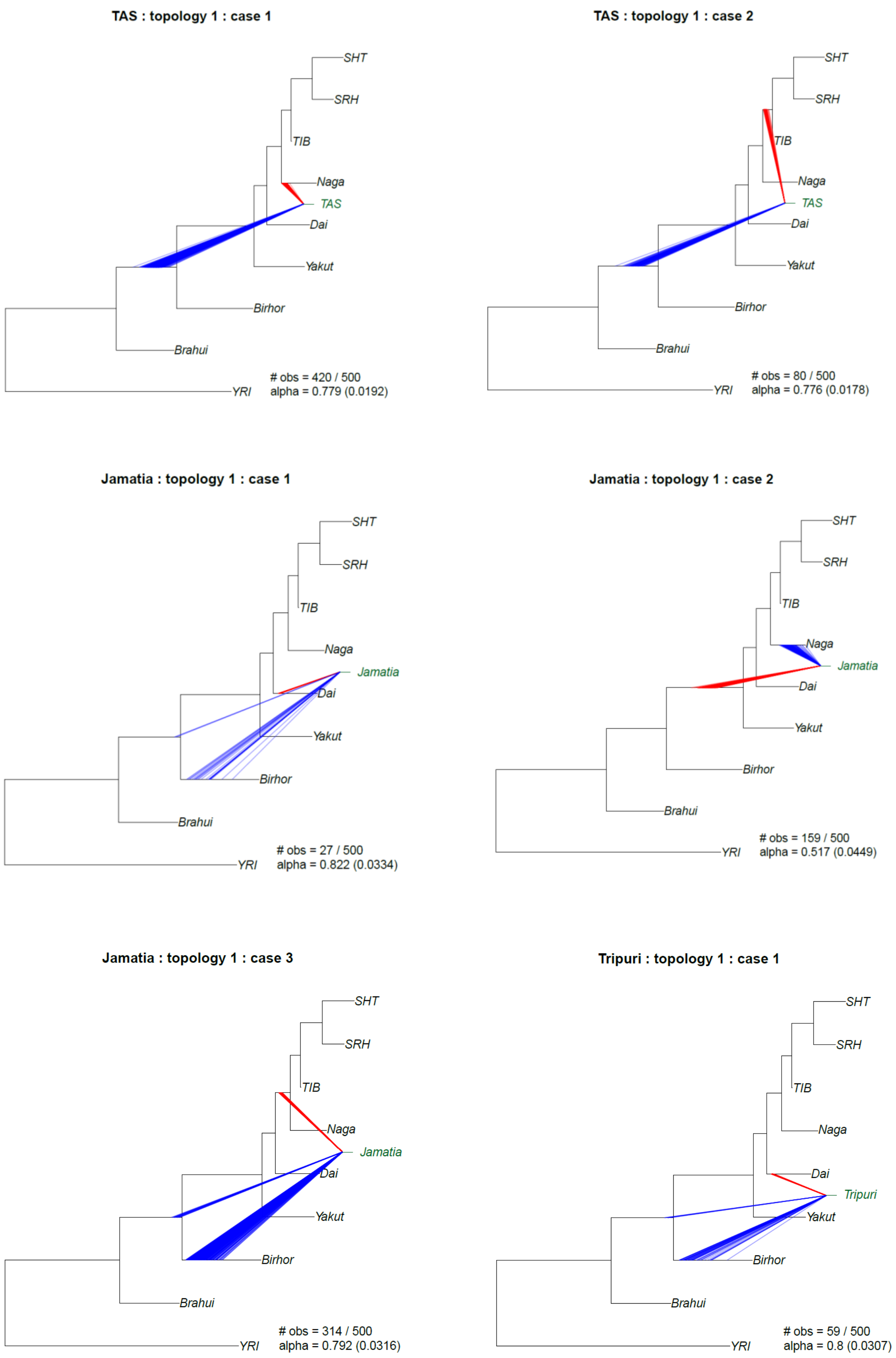
TAS : topology 1 : case 1
SHT
SRH
TIB
Naga
TAS
Dai
Yakut
Birhor
Brahui
YRI
# obs = 420 / 500
alpha = 0.779 (0.0192)
TAS : topology 1 : case 2
SHT
SRH
TIB
Naga
TAS
Dai
Yakut
Birhor
Brahui
YRI
# obs = 80 / 500
alpha = 0.776 (0.0178)
Jamatia : topology 1 : case 1
SHT
SRH
TIB
Naga
Jamatia
Dai
Yakut
Birhor
Brahui
YRI
# obs = 27 / 500
alpha = 0.822 (0.0334)
Jamatia : topology 1 : case 2
SHT
SRH
TIB
Naga
Jamatia
Dai
Yakut
Birhor
Brahui
YRI
# obs = 159 / 500
alpha = 0.517 (0.0449)
Jamatia : topology 1 : case 3
SHT
SRH
TIB
Naga
Jamatia
Dai
Yakut
Birhor
Brahui
YRI
# obs = 314 / 500
alpha = 0.792 (0.0316)
Tripuri : topology 1 : case 1
SHT
SRH
TIB
Naga
Dai
Tripuri
Yakut
Birhor
Brahui
YRI
# obs = 59 / 500
alpha = 0.8 (0.0307)

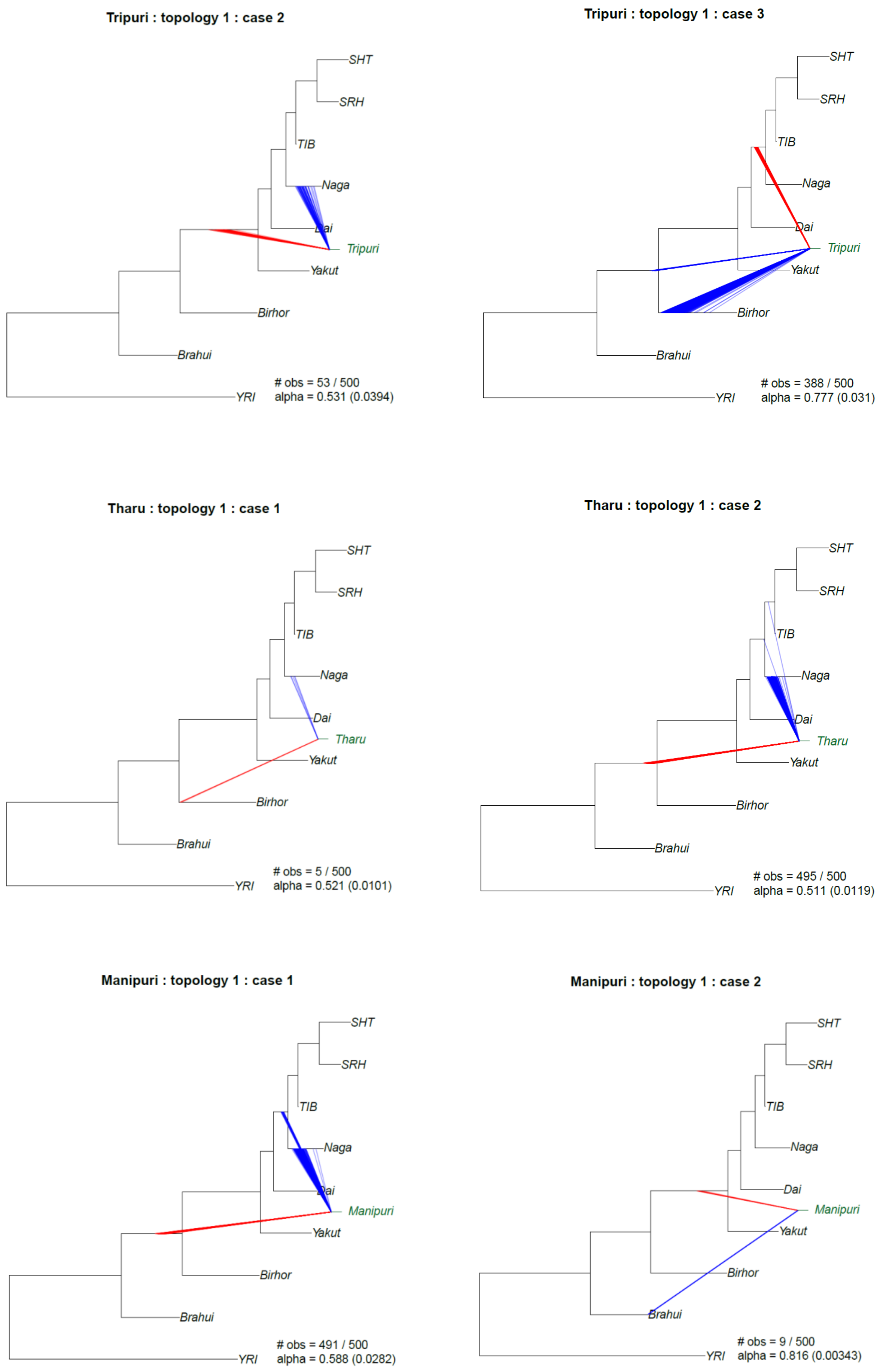
Tripuri : topology 1 : case 2
SHT
SRH
TIB
Naga
Dai
Tripuri
Yakut
Birhor
Brahui
YRI
# obs = 53 / 500
alpha = 0.531 (0.0394)
Tripuri : topology 1 : case 3
SHT
SRH
TIB
Naga
Dai
Tripuri
Yakut
Birhor
Brahui
YRI
# obs = 388 / 500
alpha = 0.777 (0.031)
Tharu : topology 1 : case 1
SHT
SRH
TIB
Naga
Dai
Tharu
Yakut
Birhor
Brahui
YRI
# obs = 5 / 500
alpha = 0.521 (0.0101)
Tharu : topology 1 : case 2
SHT
SRH
TIB
Naga
Dai
Tharu
Yakut
Birhor
Brahui
YRI
# obs = 495 / 500
alpha = 0.511 (0.0119)
Manipuri : topology 1 : case 1
SHT
SRH
TIB
Naga
Dai
Manipuri
Yakut
Birhor
Brahui
YRI
# obs = 491 / 500
alpha = 0.588 (0.0282)
Manipuri : topology 1 : case 2
SHT
SRH
TIB
Naga
Dai
Manipuri
Yakut
Birhor
Brahui
YRI
# obs = 9 / 500
alpha = 0.816 (0.00343)

Naxi : topology 1 : case 1

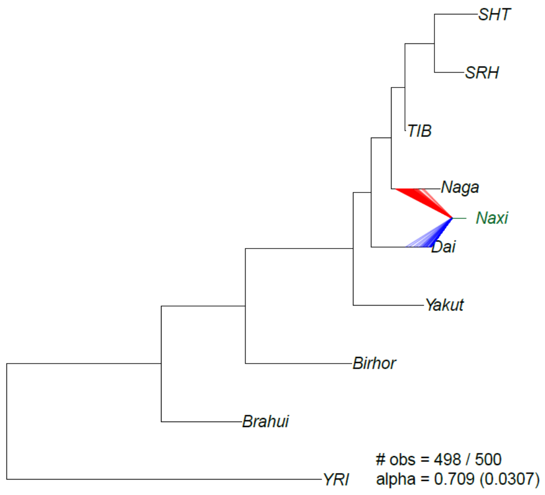

Naxi : topology 1 : case 2

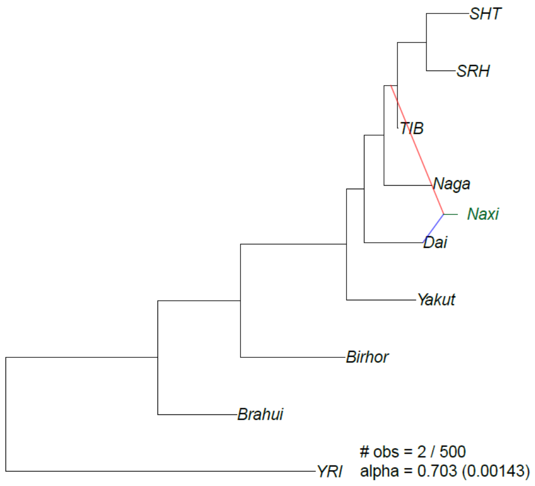

Yizu : topology 1 : case 1

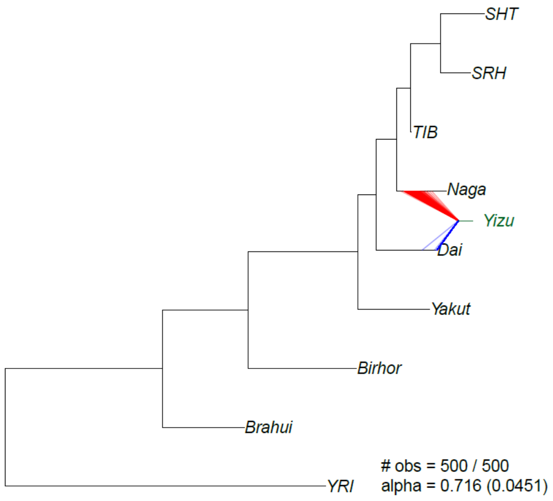

Burma : topology 1 : case 1

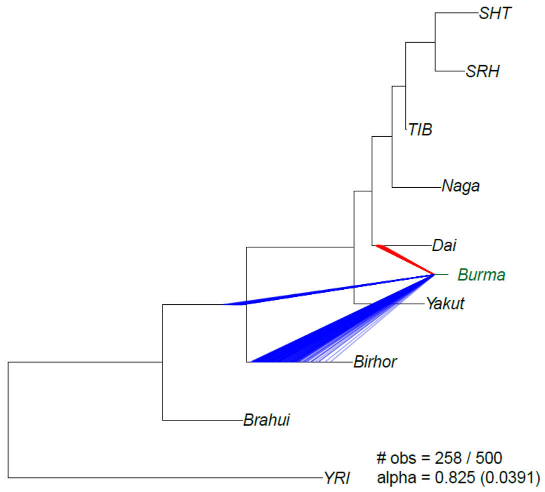

Burma : topology 1 : case 2

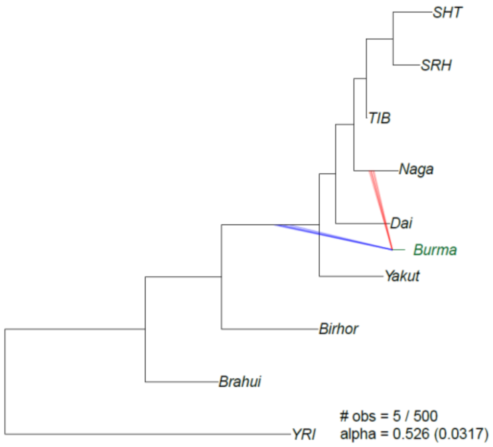

Burma : topology 1 : case 3

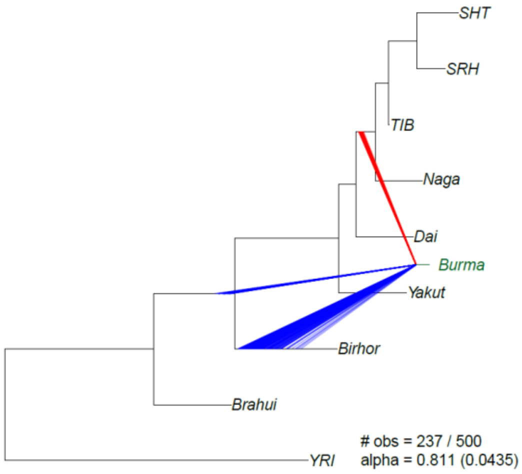

Supplementary Fig. S9. Neighbour-Joining trees showing admixture events fitted with MIXMAPPER. The main ancestry sources are represented with red lines, while the secondary sources are displayed with blue lines. These lines are located in the positions of the tree presenting the best residual norm for each one of the tested cases. Only cases with at least five observations (obs) on the 500 bootstrap replications were considered. Alpha values refers to the main sources of ancestry proportions. Topology 1 was conserved for all 500 bootstrap replications.

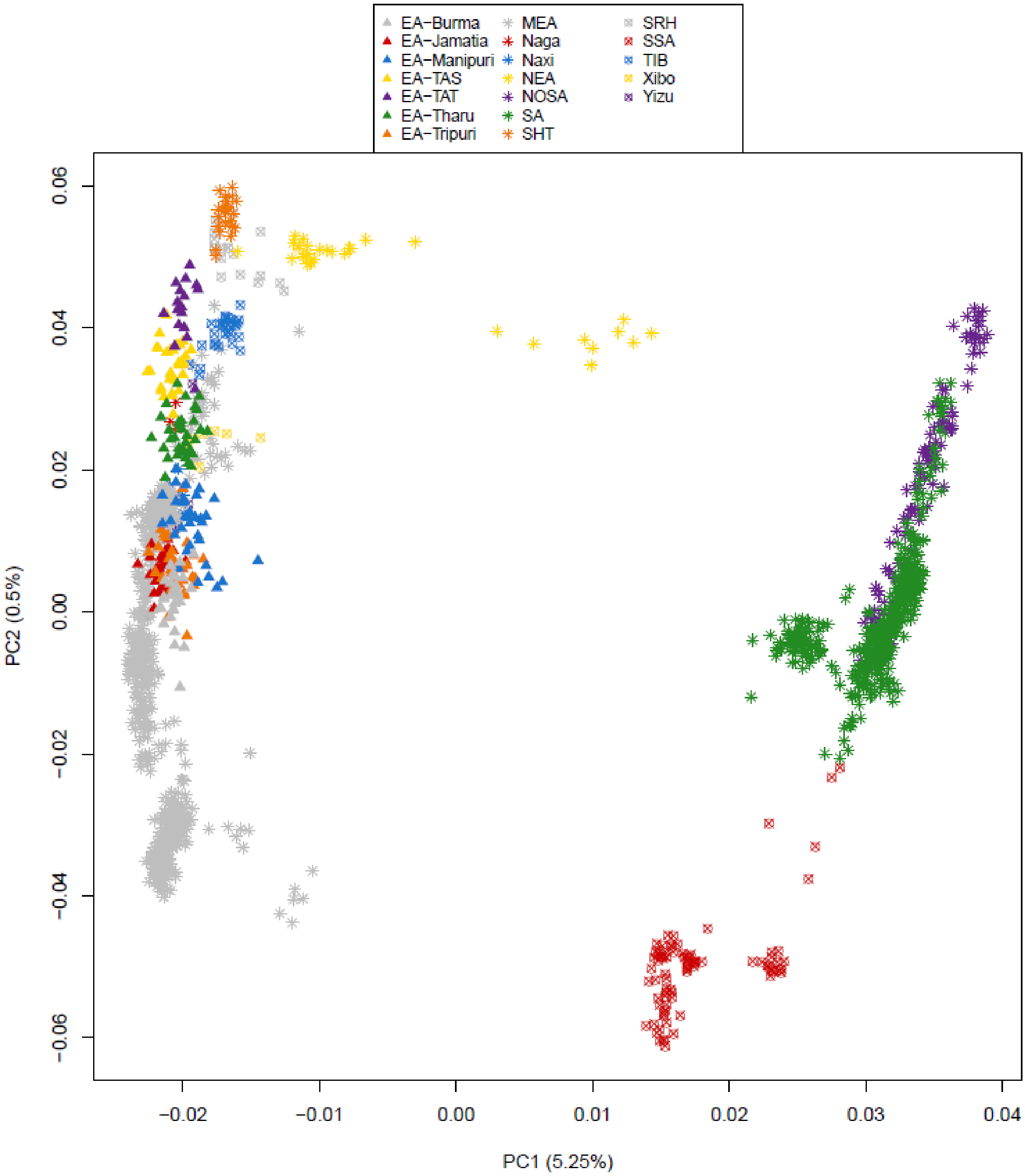

Supplementary Fig. S10. Ancestry-specific PCA performed on the 68 Asian populations included in the "extended" dataset. The seven examined EA-Tibeto-Burman populations were projected on the PCA space via the *lsqproject = YES* function implemented in the smartpca algorithm. Tibeto-Burman populations are represented with coloured triangles. The 68 EA populations are labelled according to the following macro-groups: Central EA, MEA; North EA, NEA; North SA, NOSA; South SA, SSA.

Supplementary Tables

Supplementary Tables S1-S8 are included in a separate Excel file.

Supplementary Table S1. Nepalese samples typed in the present study for ~ 720,000 genome-wide SNPs and Asian populations included in the “extended” dataset (+ YRI and CEU).

Supplementary Table S2. Significant (Z-score ≤ 2) negative *f3* values.

Supplementary Table S3. Population trios presenting significant results of ALDER analyses.

Supplementary Table S4. GCA samples typed in the present study for mtDNA HVSI sequence and Y-chromosome STRs and Asian populations included in the reference panels.

Supplementary Table S5. mtDNA HVSI and Y-chromosome STR haplotypes of the GCA and Asian reference panel populations. Number of unique haplotypes and Nei's gene diversity indices are reported for each population.

Supplementary Table S6. mtDNA and Y-chromosome haplogroup frequencies in the three GCA groups.

Supplementary Table S7. Average length of autosomal haplotype segments shared between individuals belonging to the same population as inferred by CHROMOPAINTER analysis.

Supplementary Table S8. *f4* and D-statistics computed in the form (YRI, CDX; Nagas, Tibetans/Sherpas) to test whether a single common origin between low- and high-altitude Tibeto-Burmans is consistent with available data.

Supplementary Results

Setting GCA Populations in the South/East Asian Genomic Landscape

When PCA was extended to the 75 Asian populations selected from the "extended" dataset, PC1 (4.8% of variance) captured the known genetic differentiation between groups from the SA peninsula (i.e. India and Pakistan) and from EA (i.e. Indochina, China, Japan)[1,2], while PC2 (0.6%) reflected clines of variation observable within these two macro-geographic areas. In particular, the well-established North to South gradient of SA genetic diversity[3-6] was clearly detectable. Conversely, the EA cluster appeared to be more homogeneous, with the cline described by PC2 recapitulating only to a certain degree the latitudinal extension of continental East Asia.

As regards IAR, they were substantially scattered in the PCA space, especially along PC1. For instance, two IAR individuals clustered within the Indian Tharu people, an Indo-Aryan speaking group that resides in the Terai plain and that is known to show also EA ancestry components[3,7,8]. Hereafter, we thus included Tharu in the bulk of Tibeto-Burman populations since the majority of SA groups presenting EA admixture actually speaks Tibeto-Burman languages.

Complex Admixture Patterns of GCA and Tibeto-Burman Populations

When ancestry proportions for each GCA subject were investigated via ADMIXTURE by considering a large set of SA and EA samples, IAR showed extremely variable levels of SA/EA admixture, with some individuals presenting a Northern Indian-like ancestry profile characterized by none or negligible EA ancestry and others showing significant proportions (up to 80%) of this genetic component (Fig. 2A). In contrast, GCA Tibeto-Burman populations presented homogeneous ancestry profiles. Specifically, TAS were characterized by evident SA/EA admixture, with on average 23% of their ancestry being derived from SA genetic components and 77% from EA ones, through all K presenting the highest log likelihood values. The proportion of SA ancestry was considerably lower in TAT, with a mean value of approximately 9% (Supplementary Fig. S3). Conversely, SRH showed almost no signatures of recent SA admixture, in line with what observed for the Sherpas from Khumbu.

The "Sherpa-like" component maintained relatively high proportions also in other Tibeto-Burmans. For instance, it varied from those observed in Tibeto-Burmans from the Indian subcontinent (i.e. Manipuri, 18%; Burma, 21%; Jamatia, 23%; Tripuri, 23% and Tharu, 27%) or China (i.e. Naxi, 30%; Yizu, 32%) to those proper of Nagas (42%), Tamangs (TAS, 42%; TAT, 52%) and TIB (58%), being instead detected at substantially lower values in non-Tibeto-Burman EA populations. Among them, only Tu people from the Chinese Qinghai-Gansu area and speaking Mongolic languages, but

interlinked with Tibetan dialects[9], presented a proportion comparable to that observed in Tibeto-Burmans (26%).

ALDER analysis (Supplementary Table S3; Supplementary Fig. S5) showed that only Burma, Yizu, Naxi and TIB failed to be confirmed as admixed thus representing the sole exceptions together with TAT, who turned out to be not admixed according to the computed *f3* statistics, but resulted significantly admixed with ALDER. Results obtained for the Indian Tibeto-Burman groups (i.e. Jamatia, Tripuri, Manipuri, and Tharu) were in line with those previously described by Basu et al.[3] and achieved using a different dating method. We considered a value of 25 years per generations to derive the time since admixture from the rate of exponential LD decay. Jamatia and Tharu indeed presented the oldest time ranges, spanning from 1.6 to 0.6 thousand years ago (kya), while we identified more recent admixture events for Manipuri and Tripuri (0.8-0.2 kya). As regards TAS and TAT, the inferred dates of admixture were even more recent (0.65-0.38 kya and 0.43-0.13kya, respectively).

Disentangling the Impact of Admixture and Drift on the History of Sherpa People

By estimating haplotype diversity of mtDNA (H = 0.898) and Y-chromosome (H = 0.942) in SRH and in previously studied Sherpa communities (H = 0.906 for mtDNA)[10], we found values appreciably reduced with respect to the average computed for other Tibeto-Burman, SA and EA groups (H = 0.973 for mtDNA and H = 0.980 for Y-chromosome) (Supplementary Table S4 and Supplementary Table S5). Accordingly, proportions of haplotypes shared among individuals belonging to the same population were substantially higher in Sherpa communities (SHR, 54% and 32% for mtDNA and Y-chromosome respectively; SHT, 85% for mtDNA) when compared to the mean values calculated for the assembled reference datasets (i.e. 27% for mtDNA and 18% for Y-chromosome). A pattern of remarkable intra-population homogeneity emerged also from the analysis of SRH haplogroup composition, which highlighted a limited number of lineages accounting for the great majority of the examined mtDNAs (cumulative percentage of A, C4a3b and M9a1a of 56%) and Y-chromosomes (O3, 85%) (Supplementary Table S6). These findings were in agreement with those described by Bhandari et al.[10] and corroborated the hypothesis that historical isolation and subsequent drift have actually influenced patterns of uniparental variation in Sherpa populations.

Our ROH analyses provided a first clue supporting an appreciable impact of these demographic events also on the autosomal genome of Sherpa people, especially as regards SHT who presented remarkable values for both the average length and the number of DNA segments in homozygosity (Supplementary Fig. S6).

According to these findings, we aimed at testing whether the “Sherpa-like” ancestry component pointed out by ADMXTURE was influenced by strong genetic drift experienced by the Sherpas. For this purpose, we applied a procedure based on CHROMOPAINTER “chunk-lengths” output on a subset of EA populations included in the “extended” dataset from which we excluded SHK (since they appeared to be admixed with TIB and other SA/EA populations, Supplementary Table S2) and TAT (due to their low sample size). The rationale beyond the two conducted CHROMOPAINTER runs was that if Himalayan populations would share the same painting profiles it would mean that most of the differences observable between the examined groups are ascribable to recent events of drift and gene flow that differentiated the two groups. These two sets of painting profiles were then used to infer proportions of the genome that the tested population derived from all donor groups, as described in Materials and Methods.

Genomic Relationships Between GCA and South/East Asian Populations

We calculated a series of *ad hoc* D-statistics by testing separately the three Tibeto-Burman GCA groups and each EA population and by considering a four-population phylogeny in the form: (EA, TIB; GCA, YRI). In particular, by forcing GCA groups to branch out before the split between TIB and the rest of EA populations, we aimed at testing whether the poor fit of GCA samples in such an artificial phylogeny deviates towards their closer genetic connection with TIB or with other EA groups. Accordingly, negative D values indicated high affinity with TIB, whereas positive values suggested close relationship with the tested EA population. Overall, these analyses (Supplementary Fig. S8) validated results obtained by computing outgroup *f3* statistics (Supplementary Fig. S7).

The Admixed Phylogeny of Tibeto-Burman Populations

To reconstruct Tibeto-Burman phylogenetic relationships by taking into account their SA/EA admixture profiles, we used relatively non-admixed populations identified according to *f3* statistics to build a scaffold tree by means of the MIXMAPPER algorithm. In detail, we selected Nagas, SHT and SRH as representative Tibeto-Burman groups. We then included in the tree also TIB, which presented significantly negative *f3* values, but not when tested against the other low-altitude EA populations used to build the scaffold tree. In fact, we aimed at considering also the TIB sample because we were mainly interested in identifying the branching point between low- and high-altitude Tibeto-Burmans. Nevertheless, it is important to note that our dataset included only one Tibetan population, which could be not representative of the overall genetic variation present across the plateau. We then selected the non-admixed EA population of Dai as a proxy for groups with predominant Southern EA ancestry and the Yakuts as representative of people with Northern EA

ancestry. Moreover, the Brahui and Birhor SA populations were also included to represent the two main sources of Indian ancestry (i.e. the ancestral northern and the ancestral southern ones)[3,6], together with YRI that were used as outgroup.

Although robustness of the reconstructed topology was strongly supported by 500/500 bootstrap replications, it is important to note that when formally tested via computation of *f4* and D-statistics in the form (YRI, CDX; Nagas, Tibetans/Sherpas), the divergence between Nagas and the high-altitude clade cannot be explained by a single split from a common ancestor. In fact, significantly negative *f4* and D-statistics were obtained for all tests (Supplementary Table S7), suggesting that Tibetans and Sherpas may represent a present-day proxy for a distinct branch of ancestral Tibeto-Burmans.

Isolating the East Asian Ancestry of Tibeto-Burman Populations

In a first explorative PCA performed on masked haploid data obtained by HAPMIX analysis, Central Siberian populations, such as Yakuts and Uygurs, were identified as genetic outliers, in line with their known ancient connection with a Western Eurasian ancestry source[11]. After removing such populations, we focused on those laying on the gradient described by PC1 and we noted that Tibeto-Burmans diverged from the classical South to North cline of variation present in EA, forming a distinct cluster.

Outgroup *f3* statistics computed on the masked dataset (Fig. 4B) pointed out more recent genetic relationships between Tibeto-Burmans. In fact, Tharu form the Terai plains of Northern India and Nepal resulted more closely related to TAS (*f3* = 0.220) as well, in accordance to their geographic proximity, but again with Nagas being responsible for the second highest *f3* peak (0.219). In addition, Jamatia and Tripuri showed closer affinity with each other (*f3* = 0.221) as they both reside in the Tripura region and speak sub-lineages of the Kok Borok branch of Tibeto-Burman languages[9], and then to Nagas (*f3* = 0.219 and 0.218, respectively). Instead, Manipuri turned out to be more closely related directly to Nagas (*f3* = 0.219), as expected according to their linguistic affiliation to the Kuki-Chin-Naga Tibeto-Burman branch[9].

Supplementary Materials and Methods

Data Curation

Analyses of uniparental haplotypes were carried out by combining newly generated data with those publicly available for several SA and EA populations (i.e. 48 as concerns mtDNA and 25 for Y-chromosome) (Supplementary Table S4). Nei's genetic diversity (H) was estimated with ARLEQUIN v.3.5.2.2[12], while haplogroup prediction was performed using the online HAPLOGREP2.0[13,14] and HAPLOGROUP PREDICTOR[15] tools.

As QC performed on genome-wide data, we retained only autosomal loci with a genotyping success rate higher than 95% and no significant deviations from the Hardy-Weinberg equilibrium ($p > 1.42x10^{-8}$ after Bonferroni correction for multiple testing). Moreover, one individual showing more than 5% of missing data was excluded, together with ambiguous strand SNPs with A/T or G/C alleles. The obtained high-quality dataset was submitted to LD pruning and the identified 102,980 SNPs in approximate linkage equilibrium with each other ($r^2 < 0.2$) were used to estimate the degree of recent shared ancestry (IBD) for each pair of subjects by calculating the genome-wide proportion of shared alleles (i.e. identity by state, IBS). Accordingly, we removed 12 individuals showing IBD kinship coefficient higher than 0.2. As a final QC step, we also applied PCA on the LD-pruned dataset to further exclude outliers that did not cluster with the majority of subjects belonging to their self-assigned ethnic group. Three individuals were thus filtered out as exceeding ± 3 standard deviations from the mean PC1 or PC2 eigenvector calculated for their respective cluster (Supplementary Fig. S1).

Tests Aimed at Providing Demographic Inferences

According to the equation adopted for MIXMAPPER analysis, it was possible to derive the split point of the two putative ancestral branches, the combined terminal branch length and the related mixture fraction. In particular, for each choice of the branches, the algorithm found the least-square solution from which it was possible to identify the combination of previous parameters presenting the smallest residual norm.

In order to formally test whether the clade including low- and high-altitude Tibeto-Burman groups (as resulted from MIXMAPPER analysis) is consistent with a single split of these two lineages from a common ancestor, we computed *f4* and D-statistics in the form (YRI, CDX; Nagas, Tibetans/Sherpas). For this purpose, we used the functions implemented in the ADMIXTOOLS v3.0 package and we applied default parameters (Supplementary Table S7).

Supplementary References

1. Li, J. Z. *et al.* Worldwide human relationships inferred from genome-wide patterns of variation. *Science* **319,** 1100–1104 (2008).
2. Mapping Human Genetic Diversity in Asia. *Science* **326,** 1541 (2009).
3. Basu, A., Sarkar-Roy, N. & Majumder, P. P. Genomic reconstruction of the history of extant populations of India reveals five distinct ancestral components and a complex structure. *Proc. Natl. Acad. Sci. U.S.A.* **113,** 1594–1599 (2016).
4. Chaubey, G. *et al.* Population genetic structure in Indian Austroasiatic speakers: the role of landscape barriers and sex-specific admixture. *Mol. Biol. Evol.* **28,** 1013–1024 (2011).
5. Metspalu, M. *et al.* Shared and unique components of human population structure and genome-wide signals of positive selection in South Asia. *Am. J. Hum. Genet.* **89,** 731–744 (2011).
6. Reich, D., Thangaraj, K., Patterson, N., Price, A. L. & Singh, L. Reconstructing Indian population history. *Nature* **461,** 489–494 (2009).
7. Chaubey, G. *et al.* Unravelling the distinct strains of Tharu ancestry. *Eur. J. Hum. Genet.* **22,** 1404–1412 (2014).
8. Fornarino, S. *et al.* Mitochondrial and Y-chromosome diversity of the Tharus (Nepal): a reservoir of genetic variation. *BMC Evol. Biol.* **9,** 154 (2009).
9. Paul, L. M., Simons, G. F., Fennig, C. D. *Ethnologue: Languages of the World, Nineteenth edition* http://www.ethnologue.com (2016).
10. Bhandari S, Zhang X, Cui C, Bianba null, Liao S, Peng Y, et al. Genetic evidence of a recent Tibetan ancestry to Sherpas in the Himalayan region. Sci Rep. 2015;5:16249.
11. Raghavan, M. *et al.* Upper Palaeolithic Siberian genome reveals dual ancestry of Native Americans. *Nature* **505,** 87–91 (2014).
12. Excoffier, L. & Lischer, H. E. L. Arlequin suite ver 3.5: a new series of programs to perform population genetics analyses under Linux and Windows. *Mol Ecol Resour* **10,** 564–567 (2010).
13. van Oven, M. & Kayser, M. Updated comprehensive phylogenetic tree of global human mitochondrial DNA variation. *Hum. Mutat.* **30,** E386-394 (2009).
14. Kloss-Brandstätter, A. *et al.* HaploGrep: a fast and reliable algorithm for automatic classification of mitochondrial DNA haplogroups. *Hum. Mutat.* **32,** 25–32 (2011).
15. Athey, W. T. Haplogroup prediction from Y-STR values using a Bayesian-allele-frequency approach. *J. Genet. Geneal*. **2,** 34-39 (2006).
